# The nanosilica hazard: another variable entity

**DOI:** 10.1186/1743-8977-7-39

**Published:** 2010-12-03

**Authors:** Dorota Napierska, Leen CJ Thomassen, Dominique Lison, Johan A Martens, Peter H Hoet

**Affiliations:** 1 Unit of Lung Toxicology, Katholieke Universiteit Leuven, Herestraat 49, 3000 Leuven, Belgium; 2 Center for Surface Chemistry and Catalysis, Katholieke Universiteit Leuven, Kasteelpark Arenberg 23, 3001 Heverlee, Belgium; 3 Louvain centre for Toxicology and Applied Pharmacology (LTAP), Université Catholique de Louvain, Avenue E. Mounier, 53.02, 1200 Brussels, Belgium

## Abstract

Silica nanoparticles (SNPs) are produced on an industrial scale and are an addition to a growing number of commercial products. SNPs also have great potential for a variety of diagnostic and therapeutic applications in medicine. Contrary to the well-studied crystalline micron-sized silica, relatively little information exists on the toxicity of its amorphous and nano-size forms. Because nanoparticles possess novel properties, kinetics and unusual bioactivity, their potential biological effects may differ greatly from those of micron-size bulk materials. In this review, we summarize the physico-chemical properties of the different nano-sized silica materials that can affect their interaction with biological systems, with a specific emphasis on inhalation exposure. We discuss recent *in vitro *and *in vivo *investigations into the toxicity of nanosilica, both crystalline and amorphous. Most of the *in vitro *studies of SNPs report results of cellular uptake, size- and dose-dependent cytotoxicity, increased reactive oxygen species levels and pro-inflammatory stimulation. Evidence from a limited number of *in vivo *studies demonstrates largely reversible lung inflammation, granuloma formation and focal emphysema, with no progressive lung fibrosis. Clearly, more research with standardized materials is needed to enable comparison of experimental data for the different forms of nanosilicas and to establish which physico-chemical properties are responsible for the observed toxicity of SNPs.

## Introduction

Over the past decade, the definition of nanoparticles has been controversial. **Nanoparticles **are commonly defined as objects with a diameter less than 100 nm, but no clear size cut-off exists, and this usual boundary does not appear to have a solid scientific basis. Other definitions of nanoparticles have been proposed, and the most recent proposal [1] is based on surface area rather than size (a nanoparticle should have specific surface area > 60 m^2^/cm^3^), thus reflecting the critical importance of this parameter in governing the reactivity and toxicity of nanomaterials. Physico-chemical properties that may be important in understanding the toxic effects of nanomaterials include primary particle size, agglomeration/aggregation state, size distribution, shape, crystal structure, chemical composition, surface chemistry, surface charge, and porosity. Aspects of these properties have been discussed in several reviews of nanotoxicology [2-4].

**Silica **is the common name for materials composed of silicon dioxide (SiO_2_) and occurs in crystalline and amorphous forms. Crystalline silica exists in multiple forms. Quartz, and more specifically α-quartz is a widespread and well-known material. Upon heating, α-quartz is transformed into β-quartz, trydimite and cristobalite. Porosil is the family name for porous crystalline silica. Quartz exists in natural and synthetic forms, whereas all porosils are synthetic. Amorphous silica can be divided into natural specimens (e.g., diatomaceous earth, opal and silica glass) and human-made products.

The application of synthetic amorphous silica, especially silica nanoparticles (SNPs), has received wide attention in a variety of industries. SNPs are produced on an industrial scale as additives to cosmetics, drugs, printer toners, varnishes, and food. In addition, nanosilica is being developed for a host of biomedical and biotechnological applications such as cancer therapy, DNA transfection, drug delivery, and enzyme immobilization [5-9]. Barik et al. [10] recently reviewed the impact of nanosilica on basic biology, medicine, and agro-nanoproducts. With the growing commercialization of nanotechnology products, human exposure to SNPs is increasing, and many aspects related to the size of these nanomaterials have raised concerns about safety [11]. Until recently, most research has focused on silica particles 0.5 to 10 μm, mainly in crystalline forms, but nanosilica may have different toxicological properties as compared with larger particles. The unique physico-chemical properties of nano-sized silica that make them attractive for industry may present potential hazards to human health, including an enhanced ability to penetrate intracellular targets in the lung and systemic circulation.

Biocompatibility is a critical issue for the industrial development of nanoparticles [12,13]. Even though no acute cytotoxicity has been observed or reported, the uptake of the nanoparticles by cells may eventually lead to perturbation of intracellular mechanisms. The ability of silica-coated nanomaterials to penetrate the blood-brain barrier also strongly suggests that extensive studies are required to clarify the potential chronic toxicity of these materials [14].

A number of SNPs have recently been shown to cause adverse health effects *in vitro *and *in vivo *(discussed later in this review). However, most of the studies have used poorly characterized particles in terms of their composition and physico-chemical properties. The distinct physico-chemical properties of nanoparticles indeed determine their interaction with the cell/within the cell, and even subtle differences in such properties can modulate the toxicity and modes of action. The results of toxicity studies then become difficult to interpret and compare, and, as a result, drawing appropriate conclusions is nearly impossible. Although SNPs could certainly provide benefits to society, their interaction with biological systems and potential toxic effects must be carefully addressed.

In this review, we discuss silica materials with a special attention to the physico-chemical properties that can affect their potential interaction with biological systems. We aim to provide an overview of the recent *in vitro *and *in vivo *investigations of the toxicity of nanosilica, both in crystalline and amorphous forms, rather than review the toxicity of micron-sized silica and quartz. A summary of the present knowledge on the potential toxic effects of nano-sized silica particles is needed, because their toxicological pattern appears distinct from that of micron-sized silica particles.

## Synthesis & Characterization of Silica Materials

### Classification of natural and synthetic silica materials

"Silica" is the name given to materials with the chemical formula of silicon dioxide, SiO_2_. Silicas can be amorphous or crystalline, porous or non-porous (dense), anhydrous or hydroxylated [15], regardless of their natural or synthetic nature. In a silica material, the silicon atom is in tetrahedral coordination with 4 oxygen atoms. Theoretically, an infinite variety of 3-D-ordered structures can be built from oxygen-sharing silicate tetrahedra. The number of known crystalline silica materials is limited, which leaves much room for research and development. In amorphous silica, the tetrahedra are randomly connected.

In nature, amorphous silica can have different origins. Silica can be condensed from vapors emitted in volcanic eruptions. Natural silica can also be deposited from supersaturated natural water or polymerized in living organisms (biogenic silica). These amorphous biogenic silicas can be found as isolated particles, skeletal structures or surface elements in different living organisms. Many microcrystalline silica minerals such as flint, chert and chalcedony are derived from biogenic silica after crystallization by compaction. Kieselguhr (diatomaceous earth) occurs at various stages of transformation [15] and therefore often exhibits both crystalline and amorphous silica constituents.

### Physico-chemical characteristics of synthetic silica materials related to toxicity

The silica materials presenting a toxicological hazard to human health are mainly synthetic materials and natural quartz. The physico-chemical properties of silica materials largely depend on the synthetic procedures used for their preparation. Therefore, we will briefly discuss silica synthesis processes.

### Silica synthesis

Silica is mainly synthesized from an aqueous solution, with dissociated monomeric silicic acid, Si(OH)_4_, or from a vapor of a silicon compound such as silicon tetrachloride.

**Figure 1:**
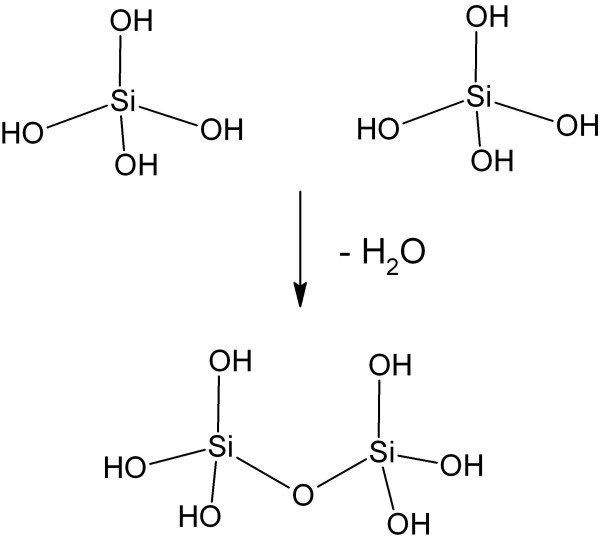
**Polymerization of silicic acid molecules through formation of siloxane bond and water**.

Waterglass is a concentrated alkaline sodium silicate solution with anhydrous composition corresponding to Na_2_SiO_3_. It is the most common reagent for silica production in aqueous solution. Waterglass is a sodium salt of silicic acid that forms silicic acid upon acidification. When the concentration of Si(OH)_4 _exceeds about 2.10^-3 ^M, condensation to polysilicic acids (Figure 1) occurs, thus leading to the formation of colloidal silica particles [15].

The polymerization and the formation of silica can be represented as follows:

[Si_n_O_2n-nx/2_(OH)_nx_] + m Si(OH)_4 _→

[Si_n+m_O_2n-nx/2+2m(2-p)_(OH)_nx+4(m-p)_] + 2 pm H_2_O

Where:

n = number of silicon atoms in a polysilicic acid molecule or particle,

x = number of OH groups per silicon atom in the polymer (0≤ × ≤ 3),

m = number of monomeric silicic acid molecules added to the polymer, and

p = fraction of the hydroxyl groups per monomeric silicic acid molecule that are converted to water during the polymerization reaction [15].

**Figure 2:**
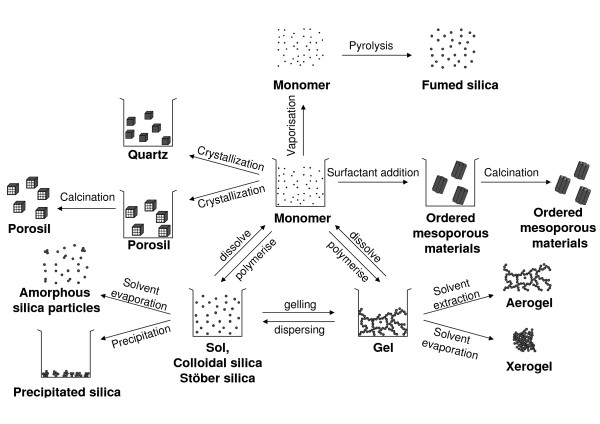
**General scheme of silica synthesis processes**. Adapted and complemented from [177].

Amorphous silica particles are formed by polymerization of monomers in the aqueous solution supersaturated with silicic acid. Various silica materials are produced in liquid phase processes (Figure 2).

**Colloidal silica **or silica sol is most often produced in a multi-step process in which the alkaline silicate solution is partially neutralized with a mineral acid. Alternatively, this pH neutralization can be achieved by electrodialysis. The resulting silica suspension is stabilized by pH adjustment. Finally a solid concentration up to 50 wt% is reached by water evaporation. Silica sol nanoparticles show a perfect spherical shape and identical size as a result of extensive Ostwald ripening [15]. **Stöber silica **sol is prepared by controlled hydrolysis and condensation of tetraethylorthosilicate (TEOS) in ethanol to which catalytic amounts of water and ammonia are added. The Stöber procedure can be used to obtain **monodisperse spherical amorphous **silica particles with tunable size and porosity [16].

**Silica gel **is obtained by destabilizing silica sol. Silica gel is an open 3-D network of aggregated sol particles. The pore size is related to the size of the original silica sol particles composing the gel.

**Precipitated silica **is formed when a sol is destabilized and precipitated.

**Ordered mesoporous silica **is obtained by a supramolecular assembly of silica around surfactant micelles. Typical surfactant molecules are amphiphilic polymers such as tribloc copolymers or quaternary alkylammonium compounds. These organic supramolecular templates are evacuated from the mesopores, typically via a calcination step. Calcination is a controlled combustion process leading to oxidation and decomposition of the template molecules into small volatile products such as NO_x_, CO_2 _and H_2_O, which can leave the pores. The diameter of the mesopores (2-50 nm) is determined by the type of surfactant applied [17,18].

A completely different synthesis route of amorphous silica starts from SiCl_4 _in the vapor phase. Silicon tetrachloride is oxidized in a hydrogen flame at temperatures exceeding 1000°C and polymerized into amorphous non-porous SNPs. This nanopowder has very low bulk density and high specific surface area, typically 200 to 300 m^2^/g. This material is called **pyrogenic or fumed silica**, referring to the special synthesis conditions [15].

The synthesis of dense crystalline silica such as **quartz **from aqueous solution is a slow process requiring heating the solution to accelerate the formation process in a so-called hydrothermal synthesis [15]. Alternatively, under high pressure, amorphous silica can be transformed to crystalline material by microcrystallization. The appearance of quartz ranges from macroscopic crystals to microcrystalline powders. Large crystals are grown at high temperature and pressure in industry. Smaller quartz crystals are conveniently obtained by grinding large crystals. Alpha-quartz is formed under moderate temperature and pressure conditions and is the most abundant form of quartz. At temperatures exceeding 573°C, α-quartz can transform into β-quartz [19]. At atmospheric pressure and temperatures higher than 870°C, quartz is transformed into tridymite and at temperatures more than 1470°C into cristobalite [15,20]. These high-temperature polymorphs of quartz have the same elemental composition but a different crystal structure and can persist metastably at lower temperatures.

Dense and porous crystalline materials can be distinguished by framework density. The framework density is conveniently defined as the number of tetrahedrally coordinated atoms (T-atoms) per nm^3^. For dense structures, such as quartz, tridymite and cristobalite, values of 22 to 29 T-atoms/nm^3 ^are common, whereas for porosils belonging to the zeolite material family, as few as 12.1 T-atoms/nm^3 ^are present [21]. The framework structure of a porosil is denoted with a 3-letter code. Descriptions are available in the *Atlas of Zeolite Framework Types *[22].

Porosils are crystallized in aqueous media in the presence of organic molecules that act as porogens or template molecules defining the size and shape of the pores. Their evacuation is typically achieved through calcination. Among the porosils are clathrasils and zeosils [23,24]. **Zeosils **have cages with windows or channels of a sufficiently free dimension to allow molecules to diffuse in and out, a property known as molecular sieving [25]. **Clathrasils **have cages with windows that are delineated with a 6-membered ring of SiO units, thus presenting a free aperture of barely 0.28 nm. Even a molecule as small as oxygen has no access to the cavities of a clathrasil. The organic template molecules engaged in the crystallization of a clathrasil cannot be removed easily from the pores [23,24].

When heated above 1700°C, any type of silica (amorphous or crystalline) melts. During cooling, the disordered structure is solidified, and a dense amorphous silica glass or **vitreous silica **is formed [15].

### Physico-chemical properties

The properties of silica materials considered essential for their potential toxicity are crystallinity, particle size and morphology, porosity, chemical purity, surface chemistry and solubility [26]. An overview of the properties of silica materials involved in silica toxicity is provided in Table 1.

**Table 1:** Overview of silica materials and relevant properties

Material	Nature of product	Crystallinity	Particle size	Porosity	Polarity	Purity	Applications	Ref
Colloidal silica	Sol	Amorphous	1-1000 nm	Dense	Hydrophillic	Very high	Binders, ink	[15]

Stober silica	Sol	Amorphous	10-1000 nm	Tunable porosity	Hydrophillic	Very high	Research	[16]

Precipitated silica	Powder	Amorphous	5-6 nm primary particles precipitated to 500 nm - 50 μm aggregates	Tunable porosity	Hydrophillic	Very high	Filler and performance additive	[15]

Silica gel	Powder	Amorphous	0.5 - 5 nm primary particles gelled to networks and milled to 500 μm - 6 mm aggregates	Tunable, void spaces between primary particles	Hydrophillic	Very high	Dessicant, filler and performance additive	[15]

Mesoporous silica	Powder	Amorphous	50 - 1000 nm, aggregated because of calcinations	Mesoporous	Hydrophobic	Very high	Drug delivery, catalysis, imaging	[8]

Pyrogenic silica (fumed silica)	Powder	Amorphous	2-50 nm primary particles fused to 1-250 μm aggregates	Void spaces between primary particles	Hydrophobic	Very high	Tickner, performance additive	[15]

Vitreous silica (fused silica glass)	Powder	Amorphous	50-2000 μm	Dense	Hydrophobic (grinded: hydrophilic)	Variable	Glass	[15,19]

Quartz	Powder	Crystalline	50 nm- several μm	Dense	Hydrophobic/(grinded: hydrophilic)	Variable	Geologic mineral, Piezoelectricity	[19,20]

Cristobalite	Powder	Crystalline	1 μm - several cm	Dense	Hydrophobic	Variable	Geologic mineral	[20]

Zeosils (porosil)	Powder	Crystalline	0.05-5000 μm	Porous Pore diameter: 0.4-1.2 nm	Hydrophillic/hydrophobic	Very high	Adsorbent	[25]

Clathrasils (porosil)	Powder	Crystalline	0.5-5000 μm	Porous Pore diameter: 0.2-0.3 nm	Hydrophillic/hydrophobic	Very high	Gas separation	[24]

Diatomeus earth, kieselguhr	Powder	Amorphous, partially crystalline	5-120 μm	Dense	Hydrophillic/hydrophobic	Low (90%)	Filter, filling material	[15]

#### Crystallinity

In crystalline structures such as quartz and porosils, the arrangement of atoms is ordered in all dimensions. According to the International Union of Pure and Applied Chemistry (IUPAC), the atoms must be arranged periodically with long-range order (at least 10 repeats in all directions) and produce sharp maxima in a diffraction experiment to observe x-ray diffraction (XRD) crystallinity [27]. The threshold for observing crystallinity depends on the unit cell size (size of the repeated unit in a crystal). For materials with large unit cells, such as porosils, the minimum particle size required is about 10 nanometers to observe a distinct, sharp XRD pattern. Amorphous silica may present some short-range order but lacks long-range order in 3 dimensions and does not exhibit a sharp XRD pattern. Of note, the surface of a crystal represents a discontinuity that can be seen as a defect. With the presence of a less-structured or even partially amorphous rim, crystals may behave like amorphous particles. Thus, particles with an ordering at limited-length scales or with amorphous regions may be classified as amorphous.

#### Particle size and morphology

**Figure 3:**
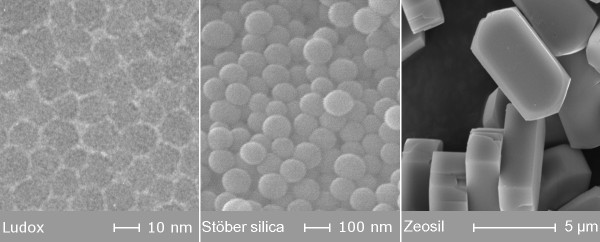
**Electron microscopy images of Stöber silica sol particles (left) and MFI type zeosil (right)**.

Nanoparticles are obtained by direct synthesis of silica sol [15] or by crystallization of nano-sized crystals of quartz or porosils [25]. The particle size is determined by the synthesis parameters. Amorphous silica sol particles tend to adopt the spherical shape so as to reach a minimum of interfacial surface area. The particle size of commercial silica sols prepared from sodium silicate is from 10 to 25 nm (Figure 3 left). Sols with larger primary particles can be prepared from TEOS by Stöber synthesis, for example (Figure 3 middle). Grinding and milling processes reduce particle size. These techniques are most often applied to quartz, silica gel and vitreous silica. The obtained products generally have a broad size distribution.

Crystalline particles exhibit crystal planes at the surface, and the morphology of the crystalline nanoparticles depends on the crystal class such as cubic, hexagonal, tetragonal, and orthorhombic (Figure 3 right). For all nanomaterials, in aqueous environment, the primary nano-sized silica particles tend to form aggregates.

#### Porosity

According to IUPAC [28], pores are classified according to their diameter into micropores (< 2 nm), mesopores (2-50 nm) and macropores (> 50 nm). Amorphous sol particles can be microporous or non-porous (dense). The porosity of Stöber silica can be tuned by adapting the synthesis parameters: decreasing the ratio of water to TEOS promotes particle growth by aggregating smaller sub-particles, thus leading to rough particle surfaces with micropores. In contrast, smooth particle surfaces are obtained with conditions of high ratio of water to TEOS [29]. Silica gel is a powder with particle size in the micrometer range or larger and is, typically, mesoporous.

**Figure 4:**
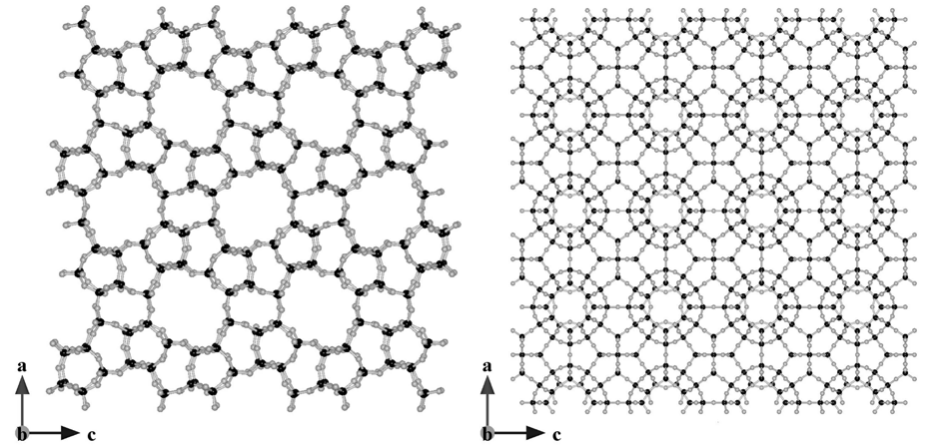
**Atomic representation of (left) a zeosil with microporous channels (MFI type) and (right) clathrasil with a denser framework (SOD type)**. Black and gray circles represent silicon and oxygen atoms, respectively. Figure made with Vesta 2.0.3 [178] with unit cell coordinates from [22].

Zeosils and clathrasils have characteristic pores and cages in the micropore size range, depending on framework topology. Examples of porosil frameworks are shown in Figure 4[22].

When the silica is presented as a nanopowder, porosity can be an intrinsic and extrinsic characteristic: stapling of the elementary nanoparticles gives rise to an interparticle porosity, which often is difficult to distinguish from the intrinsic intraparticle porosity, especially when dealing with mesoporosity.

#### Hydrophilic-hydrophobic properties

**Figure 5:**
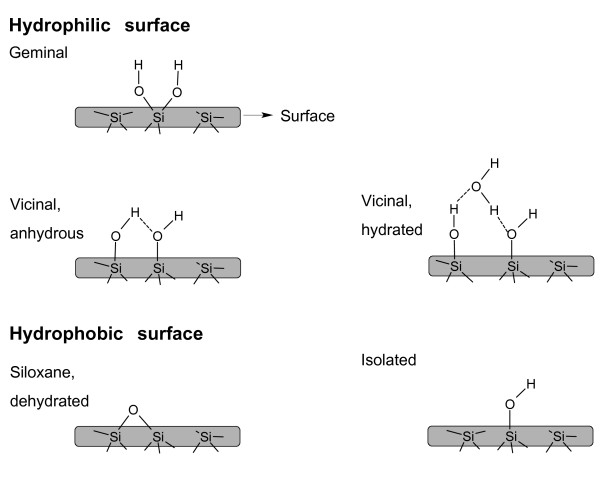
**Hydroxyl groups on the surface of silica**. Covalent bonds and hydrogen bonds are presented as full and dotted lines, respectively.

The hydrophilicity of a silica material increases with the number of silanols, or silicon-bonded hydroxyl groups, capable of forming hydrogen bonds with physical water molecules. The chemical formula of silica is represented as SiO_2_.xH_2_O, in which water represents chemical water contained in silanol groups present on the surface of the silica material. These water molecules are not to be confused with crystal water, such as that present in many inorganic salt crystals. The surface chemistry of silica is depicted in Figure 5. Vicinal hydroxyl groups (one hydroxyl group per tetrahedron) located at mutual distances smaller than 3 nm are engaged in hydrogen bonding. Geminal hydroxyls (2 hydroxyl groups per tetrahedron) are considered to occur in minor concentrations. Isolated silanols are positioned too far apart to be engaged in hydrogen bonding. Because of the differing chemistry of these 3 types of silanol groups, they are not all equivalent in their adsorption behavior or chemical reactivity. Vicinal hydroxyls interact strongly with water molecules and are responsible for the excellent water adsorption properties of silica, which are exploited in industrial gas drying operations, for example.

The reported concentration of hydroxyl groups per square nanometer on the surface of amorphous silica ranges from 4 to 5 OH/nm^2 ^[12]. As compared with amorphous silica, the crystalline forms of silica generally contain a lower concentration of surface hydroxyl groups [15]. Hydrogen-bonded water molecules are removed when silica is heated at 170°C under atmospheric pressure or at room temperature under vacuum.

Colloidal silica, precipitated silica and ordered mesoporous silica and silica gel are hydrophilic because of their high concentration of silanols. Silicagel, for example, can adsorb water in quantities up to 100% of its proper weight.

Porosils typically are hydrophobic because they lack silanols in the pores of their framework. Silica produced at high temperature, such as pyrogenic and vitreous silica, or calcined at temperatures exceeding 800°C, is almost entirely dehydroxylated. In a dehydroxylation reaction, neighboring silanol groups are condensed into siloxane bonds (Figure 5 bottom) and water molecules. Some isolated silanol groups may persist on the surface [15]. Because hydrogen bonding on siloxanes is unfavorable, dehydroxylated silica is hydrophobic. Grinding of hydrophobic bulk materials such as quartz and vitreous silica induces silicon and oxygen radicals and surface charges. These charges increase the hydrophilic surface [19,30].

#### Solubility

The dissolution and precipitation of silica in water chemically involves hydrolysis and condensation reactions, respectively, catalyzed by OH^- ^ions (Figure 1).

For micrometer-sized nonporous amorphous silica, the equilibrium concentrations of Si(OH)_4 _at 25°C in water corresponds to 70 ppm at pH 7. The silica solubility depends on the surface curvature of the (nano)particles. SNPs and nanoporous silica show enhanced equilibrium solubility, of 100-130 ppm [12]. According to Vogelsberger et al. [31], the solubilization of amorphous SNPs in physiological buffer at 25°C is accelerated because of the large surface area exposed. The solubility equilibrium is reached only after 24 to 48 h. Crystalline silica such as quartz has a much lower equilibrium solubility, of 6 ppm [15].

In summary, when dealing with silica, the physico-chemical properties such as amorphous versus crystalline nature, porosity, particle size and degree of hydroxylation must be specified. An overview of silica materials described in the scientific literature and in the research and development environment is provided in Table 1.

## Toxicity Of Silica

### Background

#### Health effects of silica and epidemiological studies

Until recently, toxicological research into silica particles focused mainly on "natural" crystalline silica particles of 0.5 to 10 μm (coarse or fine particles). This research was/is fed by the clear association of occupational inhalation exposure and severe health effects, mainly on the respiratory system. The typical lung reaction induced by chronic inhalation of crystalline silica is silicosis, a generally progressive fibrotic lung disease (pneumoconiosis), exemplified by the development of silicotic nodules composed of silica particles surrounded by whorled collagen in concentric layers, with macrophages, lymphocytes, and fibroblasts in the periphery. Epidemiologic studies have found that silicosis may develop or progress even after occupational exposure has ended; therefore, above a given lung burden of particles, silicosis was suggested to progress without further exposure [32-34]. Calvert et al. [35] recently reported an association of crystalline silica (mainly quartz) exposure and silicosis, as well as lung cancer, chronic obstructive pulmonary disease (COPD), and pulmonary tuberculosis. The carcinogenicity of quartz and cristobalite has been shown in several epidemiological studies [36-38]. In 1997, the International Agency for Research on Cancer (IARC) classified some crystalline silica polymorphs (quartz and cristobalite) in group 1 (sufficient evidence for the carcinogenicity to experimental animals and to humans), whereas amorphous silica (silicon dioxide without crystalline structure) was classified in group 3 (inadequate evidence for carcinogenicity) [39]. This classification has recently been confirmed [40]. Checkoway and Franzblau [41] reviewed occupational epidemiologic literature on the interrelations among silica exposure, silicosis and lung cancer and concluded that the appearance of silicosis is not necessarily required for the development of silica-associated lung cancer. Hnizdo and Vallyathan [42] suggested that chronic exposure to levels of crystalline silica dust, which does not cause disabling silicosis, may cause chronic bronchitis, emphysema, and/or small airway disease leading to airflow obstruction, even in the absence of radiological evidence of silicosis. Evidence has linked silica exposure to various autoimmune diseases (systemic sclerosis, rheumatoid arthritis, lupus, chronic renal disease), as reviewed by Steenland and Goldsmith [43]. A study by Haustein et al. [44] reported on silica-induced (silica dust) scleroderma.

Amorphous silica has been far less studied than has the crystalline form [39]. Warheit [45] briefly described the inhalation toxicity data related to amorphous silica particulates and concluded that some forms of amorphous silica are more potent in producing pulmonary effects as compared to others. He also emphasized the great need for adequate toxicological testing of many of these amorphous silicates given their importance in commerce and widespread potential for exposure. Workers exposed to precipitated or fumed silica did not exhibit pneumoconiosis [46,47], but evidence of pulmonary fibrosis was reported in workers exposed to amorphous silica dust produced as a byproduct of silicon metal production [48]. Merget et al. [49] reviewed the current knowledge of the health effects of a wide range of amorphous forms of silica in humans. The major problem in the assessment of health effects of biogenic amorphous silica is its contamination with crystalline silica. This problem applies particularly to the well-documented pneumoconiosis among diatomaceous-earth workers. Although the data are limited, a risk of chronic obstructive bronchitis disease, COPD or emphysema cannot be excluded [49]. Animal inhalation studies involving synthetic amorphous silica (colloidal silica, fumed silica and precipitated silica) showed at least partially reversible inflammation [50,51], granuloma formation and emphysema, but no progressive fibrosis of the lungs [52,53]. However, high doses of amorphous silica may result in acute pulmonary inflammatory responses, which could conceivably trigger long-term effects, despite a low biopersistence of the particles [54]. The debate on the health effects of micron-sized crystalline or amorphous silica is beyond the scope of this article. Readers are referred to other publications [35-38,41,55-57].

### Mechanisms of toxic action

As mentioned, most of the toxicological research into silica has focused on crystalline silica particles of 0.5 to 10 μm (coarse or fine particles). Despite the relatively large amount of available studies, the mechanisms of crystalline silica toxicity at the cellular and molecular levels are still unclear, and whether any single mechanism underlies all the above-mentioned diseases induced by these particles is uncertain [43]. However, severe inflammation following exposure to silica particles appears to be a common initiating step [58,59].

The crucial role of reactive oxygen species (ROS) in the inflammatory, fibrogenic and carcinogenic activity of quartz is well established [60,61]. Oxidative membrane and DNA damage are considered the most important mechanisms involved in the health effects of micron-sized crystalline silica. A few of the numerous reports clearly demonstrate these findings: ROS generated by the silica surface can induce cell membrane damage via lipid peroxidation that may subsequently lead to increased cellular permeability [62], perturbation of intracellular calcium homeostasis [63] and alterations in signaling pathways. Schins et al. and Fanizza et al. [64,65] demonstrated that respirable quartz particles induce oxidative DNA damage in human lung epithelial cells. Li et al. [66,67] demonstrated that micron-sized quartz particles induce ^.^OH generation through an iron-dependent mechanism. A close association of ^.^OH and iron ion concentration has been reported for amorphous silica particles [66,67]. The study of Ghiazza et al. [30] indicates that crystallinity might not be a necessary prerequisite to make a silica particle toxic; both quartz and vitreous silica showed stable surface radicals and sustained release of HO^. ^radicals. When tested on macrophages, vitreous silica and pure quartz showed a remarkable potency in cytotoxicity, release of nitrite and tumor necrosis factor α (TNF-α) production, suggesting a common behavior in inducing of oxidative stress [30]. Ding et al. [68] discuss the molecular mechanisms of silica-induced lung injuries with a focus on NF-kB activation, generation of cyclooxygenase II and tumor necrosis factor α (TNF-α). The review of Castranova [69] summarizes evidence that *in vitro *and *in vivo *exposure to crystalline silica results in activation of NF-kB and AP-1 signaling pathways. *In vitro *and *in vivo *animal studies, as well as investigations in humans, strongly support the role of macrophage products in the development and progression of silicosis [70]. Such products include a large panel of cytokines [71], with TNF-α seeming to determine the development of silica-induced pulmonary fibrosis [72]. In addition, recent evidence implicates interleukin 1β (IL-1β) and its activation by the NALP-3 inflammasome [73].

A large body of experimental work in the past 20 years has shown that 2 main factors seem to govern the hazardous nature of crystalline silica: particle surface reactivity and the form of silica [74]. Fenoglio et al. [75] evaluated these factors systematically, studying synthetic quartz samples differing only in size and shape. Cytotoxicity appeared to be primarily governed by the form of the particles and the extent of the exposed surface. Several studies indicate that the surface silanol groups are directly involved both in membranolysis [76-78] and in toxicity to alveolar cells [79,80]. Therefore, the distribution and abundance of silanols determines the degree of hydrophilicity (see "Physico-chemical properties of synthetic silica materials related to toxicity" described above) and seems to modulate cell toxicity [80,81]. Experimental work with respirable silica particles and the survey of published data by Bagchi [82] suggest that the toxicity of these particles is caused by the large amount of positive charges they carry. Ghiazza et al. [83] reported that formation of a vitreous phase at the surface of some commercial diatomaceous earth prevents the onset of oxidative stress effects. Donaldson and Borm [84] emphasized that the ability of quartz to generate ROS could be modified by a range of substances that affect the quartz surface, such as substances originating from other minerals. The authors concluded that the toxicity of quartz is not a constant entity and may vary greatly depending on the origin/constitution of the sample.

The origin/synthesis of SNPs plays a crucial role in determining the physico-chemical properties of these particles and, consequently, their potential interactions with biological systems. Surface area, surface morphology, surface energy, dissolution layer properties, adsorption and aggregation properties are relevant parameters. Depending on the manufacturing process, amorphous silica has a wide range of physico-chemical properties that determine its industrial application. Bye et al. [85] showed that the cytotoxic activity of different forms of amorphous silica does not depend on a crystalline silica component but, rather, is caused by surface charges and the morphologic features of particles. Synthetic amorphous silica has been the subject of dissolution testing with a simulated biological medium, and the silica dissolution rate was reported as being more rapid than the reverse precipitation rate [86]. Solubility has been defined as a key driver in the clearance mechanisms involved in amorphous silica removal from lung [87]. Warheit [45] reviewed pulmonary responses to different forms of silica and reported that cristobalite produced the greatest lung injury, quartz produced intermediate effects, and amorphous silica produced minimal effects. In terms of analytical technique, small differences in dissolution exist among these different forms of silica, and dissolution, in turn, influences pulmonary effects through the concept of persistence. In addition, components from the biological system may react with the surface of the particle. A systematic investigation of iron-containing SNPs as used in industrial fine-chemical synthesis demonstrated the presence of catalytic activity that could strongly alter the toxic action of nanoparticles [88].

On the whole, considering the great variety of silica forms, degree of crystallinity, surface state and the presence of contaminants, there is a critical need for carefully characterized standard silica samples to unravel the relationships between physico-chemical factors and toxicity, both micron- and nano-sized. The main goal of this review is to focus on the toxicity of nanosilica, which has never been properly reviewed. Moreover, nanosilica occurs mainly in amorphous forms, and the potential hazard posed by these nanomaterials cannot be simply related to, as has already been reviewed many times, studies of micron-sized crystalline materials.

### Silica nanoparticles

The growing abundance and industrial applications of nanotechnology has resulted in a recent shift of toxicological research towards nanoparticles [89-94]. Ultrafine particles (< 0.1 μm) have been demonstrated to cause greater inflammatory responses and particle-mediated lung diseases than have fine particles (< 2.5 μm) per given mass [95-97]. Also, experiments involving silica have shown that nanoparticles, both ultrafine colloidal silica [98,99] and crystalline silica [99], have a greater ability to cause lung injury as compared with fine particles. Thus, the unique properties (i.e., small size and corresponding large specific surface area; cell penetrating ability) of nano-sized SiO_2 _are likely to impose biological effects that differ greatly from micron-scale counterparts.

### *In vitro *studies of nanosilica toxicity

A structured summary of *in vitro *studies of the toxicity of SNPs can be found in Table 2.

**Table 2:** In vitro studies on nanosilica particles (SNPs) toxicity

Silica form	Size (primary)	Material characterization	Cells used	Test	Biological endpoints and findings	Ref
Amorphous	40 nm- 5 μm	Not specified	A549 HEp-2 RPMI 2650 RLE-6TN N2a	• Replication and transcription assays • Cell proliferation and cell viability assay • Proteasome activity assay • Immunofluorescence and microscopy	• Uptake of all particles into the cytoplasm and nuclear localization of nanoparticles between 40 and 70 nm • The uptake of NSPs in the nucleus induced aberrant clusters of topoisomerase I and protein aggregates in the nucleoplasm	[100]

Amorphous (luminescent)	50 nm	• Synthesis (ref. to literature)	A549 rat alveolar macrophages	• laser scanning confocal microscope • Comet Assay • Pulse Field Gel Electrophoresis (PFGE) • Western Blot Analysis of DNA Adducts/DNA Agarose Gel • DNA Repair Enzyme Activity Assay • Cell Proliferation Assay • Vybrant Apoptosis Assay	• Uptake not detected in the nuclear region • As compared to the A549 cells, the nanoparticle penetration rate was much faster in the rat alveolar macrophages • No significant toxic effects observed at the molecular and cellular levels below a concentration of 0.1 mg/ml	[101]

Amorphous (colloidal)	15 and 46 nm	• Particle sizes and distribution • Surface area (268 and 52.5 m^2^/g for 15 and 46 nm particle, respectively), crystalline structure, major trace metal impurities • Hydrodynamic particle size in water suspension	A549	• SRB (sulforhodamine B) and LDH assays • Reduced glutathione (GSH) level • DCFH assay (ROS generation) • Malondialdehyde (MDA) assay	• Cytotoxicity was dose- and time-dependent • Reduced glutathione (GSH) levels and elevated MDA production after exposure to 15 nm SNPs	[102]

Amorphous	60 and 100 nm	• Size distribution analysis • Endotoxin concentration	A549 THP-1 Mono Mac 6; co-cultures	• LDH assay • Cytokine expression (TNF**-α**, IL-6, IL-8) • Light and transmission electron microscopy (TEM)	• Cytotoxicity differed among the cell lines and was dose- and size-dependent (smaller particles were more toxic) • co-cultures showed an increased sensitivity to particles concerning the cytokine release in comparison to the mono-cultures of each cell type	[103]

Amorphous	~14 nm	• Size distribution	A549 L-132 HeLa MNNG/ HOS	• MTT and WST-1 assays • Trypan blue exclusion and LDH assay • Annexin V-PI assay (fluorescence microscopy) • DCFH assay • IL-8 expression (ELISA)	• Little cytotoxic effects in 4 cell lines tested at the concentration below 250 μg/ml within 48 h • Exposing cancer cells to high concentrations (250-500 μg/ml) for 72 h resulted in an inflammatory response with oxidative stress and membrane damage, which varied with cell type (A549>HOS > HeLa) • SNPs triggered an inflammation response without causing considerable cell death for both cancer cells and normal cells	[104]

Amorphous	10 and 80 nm	o Provided by producer for the primary particles (surface area: 640 and 440 m^2^/g for 10 and 80 nm particle, respectively) o Hydrodynamic particle size (in cell culture medium)	A549	• MTT and LDH assays • DCFH assay • Intracellular glutathione (GSH) concentration • Membrane lipid peroxidation (LPO) • Assay of glutathione reductase and glutathione peoxidase	• Cytotoxicity was dose-dependent • SNPs induced reactive oxygen species and membrane lipid peroxidation in dose-dependent manner • Both sizes of SNPs had little effect on GSH level and the activities of glutathione metabolizing enzymes	[105]

Amorphous	7 and 5-15 nm	o Surface area (350 and 644 m^2^/g for 7 and 5-15 nm particle, respectively) o Size distribution (in the test medium)	Beas-2B	• Incorporation of SNPs into the cells (confocal LSM) • MTT assay • PI staining (flow cytometry) • Apoptosis • DCFH assay • Oxidative stress responding transcription factors (Western blotting)	• SNPs were incorporated into the cells and distributed around the nucleus area • SNPs induced oxidative stress via ROS formation and induction of of antioxidant enzymes (SOD and HO-1) • Induction of Nrf-2-ERK MAP kinase signaling pathway was observed • Overall, cells exposed to 5-15 nm SNPs (porous) showed a more sensitive response than those exposed to 7 nm SNPs (fumed)	[106]

Amorphous	10-20 nm	o Provided by manufacturer (surface area: 140-180 m^2^/g) o Primary particle size o Endotoxin content (LPS)	A549	• MTT and LDH assays • DCFH assay • SOD activity determination • Nitrate/nitrite determination • DNA oxidative damage assay	• Cytotoxicity was dose- and time-dependent • SNPs stimulated the ROS generation, GSH depletion and lower expression of SOD activity in a dose-dependent manner • No NO production and significant DNA oxidative damage was observed after treatment of cells with SNPs • Co-treatment of LPS with SNPs enhanced observed cytoxicity and generation of oxidative stress	[107]

Amorphous	30, 48, 118 and 535 nm	• Synthesis method • Hydrodynamic particle size (in water and cell culture medium)	HEL-30	• MTT and LDH assays • Reduced glutathione (GSH) and DCFH assay • Transmission electron microscopy (TEM)	• Cytotoxicity was dose- and size-dependent (smaller particles were more toxic) • Uptake of all particles into the cytoplasm (nuclear uptake not studied) • GSH level reduced significantly of after exposure to 30 nm nanoparticles • No significant Reactive Oxygen Species (ROS) formation	[108]

Amorphous	70, 300 and 1000 nm	Not specified	XS52	• TEM analysis of cells • LDH assay • Proliferation ([^3^H]-Thymidine incorporation assay)	• SNPs of 300 and 1000 nm were incorporated into the cells and located in cytoplasm only; nanoparticles of 70 nm were located in nucleus as well as cytoplasm • Cell proliferation was inhibited by treatment with SNPs of all sizes in dose-dependent manner • The growth of the cells was more strongly inhibited by smaller-sized SNPs	[109]

Amorphous	15, 30 and 365 nm	• Size distribution • Zeta potential • Amorphous structure	HaCaT	• CCK assay • Cell cycle assay • Annexin V-PI assay (Flow cytometry) • 2D-DIGE and, IEF and SDS_PAGE (protein expression) • Western blot	• Cytotoxicity was dose- and size-dependent (smaller particles were more toxic) • Apoptosis was dose- and size-dependent (smaller particles induced higher apoptosis frequency) • Up-regulated proteins were classified as oxidative stress-associated proteins; cytoskeleton-associated proteins; molecular chaperones; energy metabolism-associated proteins; apoptosis and tumor-associated proteins	[110]

Amorphous	15 nm	• Size distribution • Zeta potential • Amorphous structure	HaCaT	• Flow cytometric analysis of methylated DNA • Real-time PCR • Western blot	• Treatment with SNPs induced Global DNA hypomethylation	[111]

Amorphous	21 and 80 nm	• Particle preparation and dispersion • Size, morphology and chemical states of elements • Hydrodynamic particle size (dispersed in water)	WS1 CCD-966sk MRC-5 A549 MKN-28 HT-29	• MTT and LDH assays	• Toxicity was seen at concentrations exceeding 138 μg/ml • Susceptibility to NSPs differed among tested cell lines	[113]

Amorphous	20 nm	Only provided by producer (surface area: 640 ± 50 m^2^/g)	RAW264.7	• Membrane fluidity measurements (FRAP technique by LSCM) • DCFH assay • Intracellular free calcium content	• Exposure to SNPs increased ROS generation and decrease of the membrane fluidity • Perturbation of Intracellular free calcium homeostasis was responsible for observed cytotoxicity	[114]

Amorphous	14 nm	Only provided by producer (surface area: 200 m^2^/g)	Caco-2	• LDH and WST-1 assay • Fpg-modified comet assay • Total GSH content	• Cytotoxicity observed • Oxidative DNA damage • Significant depletion of intracellular GSH	[115]

Amorphous	21, 48 and 86 nm	• Size distribution analysis • Surface area (225, 106 and 39 m^2^/g for 21, 48 and 86 nm particle, respectively) • structure	L-02	• MTT and LDH assays • TEM assay • DCFH, MDA and GSH assay • Annexin V-PI assay (flow cytometry) • DNA ladder assay • Western blot	• Cytotoxicity was dose- time - and size-dependent (smaller particles were more toxic) • 21 nm SNPs induced ROS generation, lipid peroxidation and GSH depletion in a dose-dependent manner • 21 nm SNPs induced apoptosis in a dose-dependent manner	[116]

Amorphous	4-40 nm (mean size: 14)	Not specified	HDMEC	• MTS assay • transmission electron microscopy (TEM) • Ki67 expression and IL-8 release	• The particles were internalized but they did not exert cytotoxic effects • Reduction of the proliferative activity and a pro-inflammatory stimulation were observed	[117]

Amorphous (monodisperse)	14, 15, 16, 19, 60, 104, 335 nm	• Particle preparation and stability • shape and size distribution • surface area (196, 179, 183, 145, 33, 28 and 7.7 m^2^/g for 14, 15, 16, 19, 60, 104 and 335 nm particle, respectively) • micropore volume • Hydrodynamic particle size (in water and cell culture medium)	EAHY926	• MTT and LDH assays • Annexin V-PI assay	• Cytotoxicity was dose- and size-dependent (smaller particles were more toxic and affected the exposed cells faster) • Cell death predominantly caused by necrosis	[118]

Amorphous	21 and 48 nm	• Size distribution analysis • Surface area (225 and 106 m^2^/g for 21 and 48 nm particle, respectively) • structure	H9c2(2-1)	• MTT and LDH assays • Hematoxylin and eosin staining • DCFH, intracellular MDA and GSH assays • Flow cytometry (cell cycle) • Western blot	• Cytotoxicity was dose- time - and size-dependent (smaller particles were more toxic) • ROS generation in a dose-dependent manner; increased level of MDA and decreased concentration of GSH indicated oxidative stress • Cell cycle arrest in G1 phase • Dose-dependent expression of p53 and p21 for 21 nm SNP	[119]

Amorphous	From 20 nm to below 400 nm	• the dispersion characteristics (size, size distribution, size evolution) • zeta potential	3T3-L1	• comet assay	• No detectable genotoxicity (the results were independently validated in two separate laboratories)	[120]

Amorphous (monodisperse)	16, 60 and 104 nm	• Particle preparation and stability • shape and size distribution • surface area (183, 33 and 28 m^2^/g for 16, 60 and 104 nm particle, respectively) • micropore volume • Hydrodynamic particle size (in water and cell culture medium)	A549	• MTT assay • cytochalasin-B micronucleus assay (CBMN) alone or in combination with FISH-centromeric staining • Alkaline Comet assay • Measurements of cell-associated silica (ICP-MS)	• Results suggest that non-cytotoxic doses of SNPs may be capable of inducing slight chromosome breakage, loss and mitotic slippage, and at higher concentration possibly mitotic arrest.	[122]

Amorphous (monodisperse)	from 2 up to 335 nm	• Particle preparation and stability • shape and size distribution • surface area (from 232 to 7.7 m^2^/g) • micropore volume • Hydrodynamic particle size (in water and cell culture medium) • Zeta potential	J774 EAHY926 3T3 Human erythrocytes	• MTT and WST-1 assays • RBC hemolysis	• in murine macrophages, the cytotoxic response, after treatment with SNPs of 17 different sizes, increased with external surface area and decreased with micopore volume • in human endothelial cells and mouse embryo fibroblast the cytotoxicity increased with surface roughness and decrease in diameter • the hemolytic activity of SNPs in human erythrocytes increased with the diameter of SNPs	[141]

Amorphous	30 nm	• Provided by producer for primary partilcles (surface area: 165 m^2^/g) • Hydrodynamic particle size (in PBS and cell culture medium) • Adsorption of proteins from the test media in the absence of cells	3T3 hT RAW264.7	• MTS assay • Uptake (flow cytometry) • DCFH assay • Lysosomal membrane integrity • Mitochodrial membrane potential • Apoptosis (caspase-3, and caspase-7 activation; Annexin V-PI assay)	• SNPs depleted serum proteins from cell culture media • SNPs cytotoxicity was dose-, time- and cell line dependent-dependent • SNPs induced significant ROS generation in all cell lines • No detectable destabilization of lysosomal membranes was observed • Incubation with SNPs decreased mitochodrial membrane potential in hT and RAW cells • SNPs triggered different extent of cell apoptosis depending on the cell line tested	[140]

Amorphous (mesoporous)	110 nm (pore diameter of ~2.5 nm)	• Structure • surface area (910 m^2^/g) • pore volume • stability in aqueous solution	3T3-L1 MCF-7 K562	• Confocal microscope • TEM • Flow cytometry	• Particles were internalized into cells and accumulated in cytoplasm • No apparent cytotoxicity	[123]

Amorphous (mesoporous)	Not specified (MCM-41 particle type)	• Synthesis and functionalization of particles • Zeta potential • Cylindrical pores with a diameter around 5 nm	HeLa	• MTT, WST-1 and LDH assays • Flow cytometry for PI • TEM observations	• No cytotoxicity was observed up to 50 μg/ml • Particles interfered with MTT assay	[126]

Amorphous (mesoporous)	108, 110, 111 and 115 nm	• Synthesis (ref to the previous study) and surface modification • Zeta potential • Surface area (780, 980, 930 and 1050 m^2^/g for 108, 110, 111 and 115 nm particle, respectively) • pore volume and pore size distribution (2.6-2.0 nm)	hMSCs 3T3-L1	• MTT assay • Flow cytometry for the uptake • Cellular differentiation and cytochemical assay	• The modulation of surface charge and its threshold affects the uptake and is specific to cell type • Positive correlation of positive surface charge and the uptake by the cells • Uptake was through clathrin and actin-dependent endocytosis • Uptake did not affect cells viability, proliferation and differentiation	[125]

Amorphous (mesoporous silica nanorods capped with iron oxide NPs)	200 × 80 nm (pore diameter of ~3 nm)	• Preparation and functionalization	HeLa	• Confocal fluorescence microscopy	• Particles were endocytosed by the cells and biocompatible (concentration used: 0.2 mg/mL)	[127]

Amorphous (mesoporous)	30, 50, 110, 170 and 280 nm	• Synthesis, suspension stability (no interparticle aggregation), hydrodynamic diamaters, zeta potential	HeLa	• MTT • onfocal laser scanning microscopy • ICP-MS	• Cellular uptake is highly particle size-dependent (with the optimum size of 50 nm); little cytotoxicity up to 100 mg/ml	[128]

Amorphous (mesoporous) loaded with anticancer drugs)	<130 nm (pore diameter of ~2 nm)	• Preparation, shape, aggregation/stability in aqueous solution	PANC-1 AsPC-1 Capan-1 MKN45 SW480	• Fluorescence and confocal microscopy	• The particles offer the possibility of controlled release of anticancer drugs (non-loaded particles did not caused cytotoxicity)	[129]

Amorphous (mesoporous)	150 nm (pore diameter of ~2.4 nm)	• Synthesis, functionalization, surface area (850 m^2^/g), zeta potential	HeLa	• Flow cytometry • Fluorescence microscopy	• Uptake of particles can be regulated by different surface functionalization • More negatively charged particles were able to escape from endosomes	[130]

Amorphous (mesoporous) Commercially available amorphous silica material	100 - 300 nm (pore diameter of ~3 nm) -	• Synthesis (ref. to the previous study), funcionalization, surface area (1138 m^2^/g), pore volumes, number of silanol group • Funcionalization	Rabbit RBCs	• Hemolysis assay • UV/Vis spectroscopy • Flow cytometry	• The hemolytic activity of silica nanoparticles depends only on the concentration of negatively charged silanol groups • Mesoporous particles exhibit a high compatibility towards RBCs as most of the silanols are located in the interior of the particles that are not accessible by the RBCs membranes	[131]

Amorphous (mesoporous)	300-650 nm (pore diameter of 31Å) and SBA-15 type (>hundreds of nm, pore diameter of 55 Å)	• Synthesis, • Order of mesostructures, surface area (821 and 506 m^2^/g), wall thickness, composition	HL-60 Jurkat	• Oxygen consumption assay • ATP formation assay • Cellular GSH assay	• Particles with larger size and larger pores caused concentration- and time dependent inhibition of cellular respiration • Both nanoparticles were toxic to the isolated mitochondria • No significant changes in cellular glutathione level was observed	[132]

Amorphous (mesoporous and silica nanospheres)	250 nm; 166x320 nm (pore diameter = 3.5 nm)	• Synthesis and functionalization • Number of particles per gram, surface area (4.1 and 0.2 m^2^/particle for mesoporous and spherical particle, respectively)	SK-N-SH	• Staining with trypan blue and determination of viable cells using a hemacytometer	• The cytotoxicity of particles was related to the adsorptive surface area of the particle (the most toxic malodorous silica are those with the largest BET surface areas) • Dependency of cytotoxicity on the nature of the attached functional groups cannot be ruled out	[133]

Amorphous (mesoporous)	270 ± 50 nm (pore diameter of 3.9 nm) and 2.5 μm ± 500 nm (pore diameter of 2.8 nm)	• Synthesis • The structural and textural characterizations • Surface area(520 and 547 m^2^/g for 270 nm and 2.5 μm particle, respectively) • LPS concentration analysis	Human monocyte-derived dendritic cells	• Apoptosis/necrosis (Annexin V/PI assay) • production of cytokinesIL-10 and IL-12p70,IL-12, IL-10 • confocal microscopy, TEM	• Viability, uptake and immune regulatory markers were affected with increasing size and dose	[134]

Amorphous (mesoporous)	190, 420 and 1220 nm	• Synthesis and functionalization • Size distribution • Dispersity and porosity • Surface area (220-650 m^2^/g) • Zeta potential	MDA-MB-468 COS-7	• MTT assay • The biodegradation experiments • Intracellular localization of particles	• The cytotoxicity of particles was highly correlated with particle sizes ((smaller particles were more toxic) • The biodegradation products of spherical E-MS particles showed no toxicity • The residual surfactant bound to the particles has a much smaller contribution to the cytotoxicity than the free one • The smaller particles were more easily endocytosed and consequently located within lysosomes	[135]

Amorphous	100 and 200 nm	• rod-shaped and spherical particles (Stöber), not-coated and coated with fibronectin or polyethylene glycol (PEG), • Primary and aggregate size, surface area (9.2 and 4.6 m^2^/g for silica rods and 27.3 and 14.2 m^2^/g for silica spheres), crystallinity, impurities, zeta potential	MET-5A	• LDH assay • Expression of IL-8 • Simulated stretch imposed on the cells	• Dosimetric comparison of acicular and isotropic particulate materials is not straightforward • In the absence of simulated lung function (stretch), cells showed no significant enhancement of cytotoxicity or inflammation release • PEG surface treatment tended to reduce the cytotoxicity and IL-8 release from particle exposures suggesting the significance of adhesive interactions e.g. for membrane binding/signal transduction	[136]

Amorphous	130 nm and 155 nm; iron oxide particle with silica shell (80 nm)	• Size distribution • Reference given for the description in detail	Hmy2 Jurkat U937 PC3; human peripheral blood cells	• MTT assay and Trypan Blue exclusion • Scanning electron microscopy • DCFH assay	• The cytotoxicity of particles depended on the cell type tested • No direct correlation between ROS production and cell toxicity. • PEG-ylation of SNP protected the particles from protein adsorption on the external surface of the NPs and consequently no agglomeration in culture medium was observed. • The availability of the particles to be internalized by the cells depended on the size and morphology of the aggregates.	[137]

Crystalline	Particle sizes not uniform (7.21, 9.08 and 123.21 nm)	• Size and concentration	WIL2-NS	• MTT assay • Population Growth Assay • Apoptosis Assay by Flow Cytometry • Cytokinesis Block Micronucleus Assay • Comet Assay • HPRT Mutation Assay	• Significant dose-dependent decrease in viability • with increasing dose of particles • Fourfold increase in micronucleated binucleated cells frequency was detected, while no significant difference was measured by the Comet assay	[99]

Chen and von Mikecz [100] investigated the effects of nanoparticles on structure, function, and proteasomal proteolysis in the cell nucleus by incubating different cell lines with unlabeled and fluorescently labeled **amorphous silica **particles of different sizes [100]. SiO_2 _particles between 40 nm and 5 μm were applied to epithelial cells in culture and observed on confocal laser scanning microscopy with differential interference contrast. Particles of all tested sizes penetrated the cytoplasm; however, nuclear localization was observed exclusively in cells treated with SiO_2 _nanoparticles between 40 and 70 nm. Fine and coarse SiO_2 _particles (0.2-5 μm) were exclusively located in the cytoplasm and accumulated around the nucleus, forming nuclear indentations. The uptake of SNPs in the nucleus induced aberrant clusters of topoisomerase I and protein aggregates in the nucleoplasm -- the former inhibiting replication, transcription, and cell proliferation -- without altering cell viability. Cells treated with fine (0.5 μm) or coarse (5 μm) SiO_2 _particles had the same replication and transcription activity as that of untreated control cells [100].

Jin et al. [101] investigated the potential toxicity of luminescent **amorphous **SNPs (50 nm) in freshly isolated rat alveolar macrophage cells and human lung epithelial cells (A549 cells). The SNPs penetrated the cells but were not detected in the nuclear region and did not cause significant toxic effects at the molecular and cellular levels below a concentration of 0.1 mg/ml.

Lin et al. [102] investigated the cytotoxicity of **amorphous (colloidal) **SNPs (15 and 46 nm) in cultured human alveolar epithelial cells (A549 cells). Cell viability decreased in a time- and dose-dependent manner (down to 100 μg/ml), and nanoparticles of both sizes were more cytotoxic than were fine quartz particles (Min-U-Sil 5). Exposure to 15-nm SNPs generated oxidative stress in A549 cells as reflected by reduced glutathione (GSH) levels, elevated production of malondialdehyde (MDA) and lactate dehydrogenase (LDH) leakage, which is indicative of lipid peroxidation and membrane damage, respectively [102]. In the study by Wottrich et al. [103], A549 cells and macrophages (THP-1, Mono Mac 6) exposed to 60 nm **amorphous **SNPs showed distinctly higher mortality than did larger silica particles (diameter 100 nm). Another study by Choi et al. [104], involving A549 cells and **amorphous **SNPs (14 nm), showed a pro-inflammatory response triggered by nanoparticles without blocking cell proliferation or causing cell death to any great extent. A recent work by Akhtar et al. [105] examined cytotoxicity (by MTT and LDH assay) and oxidative stress (ROS levels, membrane lipid peroxidation, GSH level and activity of GSH metabolizing enzymes) in A549 cells exposed for 48 h to **amorphous **SNPs of 10 and 80 nm. The SNPs were cytotoxic to studied cells through oxidant generation (ROS and membrane lipid peroxidation) rather than depletion of GSH. Eom and Choi [106] studied oxidative stress caused by **amorphous **SNPs (7 and 5-15 nm) in human bronchial epithelial cells (BEAS-2B) and observed the formation of ROS and induction of antioxidant enzymes.

Shi et al. [107] exposed A549 cells to **amorphous **SNPs (10-20 nm) at concentrations up to 200 μg/ml and observed low cytotoxicity as measured by MTT and LDH assays. However, co-treatment with the same nanoparticles and lipopolysaccharide, a bacterial product that may contaminate (nano)materials, significantly enhanced the cytotoxicity.

Yu et al. [108] examined the cytotoxic activity (by MTT and LDH assay) of well-dispersed **amorphous silica **particles (30-535 nm) in mouse keratinocytes. All sizes of particles were taken up into the cell cytoplasm; nuclear uptake was not studied. The toxicity was dose and size dependent, with 30- and 48-nm particles being more cytotoxic than 118- and 535-nm particles. The reduced GSH level significantly decreased only after exposure to 30-nm nanoparticles [108]. Nabeshi et al. [109] showed the size-dependent cytotoxic effects of **amorphous silica **particles (70, 300 and 1000 nm) on mouse epidermal Langerhans cells. The smallest particles induced greater cytotoxicity (by LDH assay) and inhibited cellular proliferation (by [^3^H]-thymidine incorporation). The observed effects were associated with the quantity of particle uptake into the cells.

Yang et al. [110] evaluated the effects of **amorphous **SNPs (15 and 30 nm) and micron-sized silica particles on cellular viability, cell cycle, apoptosis and protein expression in the human epidermal keratinocyte cell line HaCaT. Microscopy examination revealed morphological changes after 24-h exposure; cell growth also appeared to be significantly inhibited. The cellular viability of HaCaT cells was significantly decreased, and the amount of apoptotic cells was increased in a dose-dependent manner after treatment with nano- and micron-sized SiO_2 _particles. Furthermore, smaller silica particles were more cytotoxic and induced a higher apoptotic rate. Proteomic analysis revealed differential induction of expression of 16 proteins by SiO_2 _exposure; proteins were classified into 5 categories according to their functions: oxidative stress-associated proteins, cytoskeleton-associated proteins, molecular chaperones, energy metabolism-associated proteins, and apoptosis and tumor-associated proteins. The expression levels of the differentially expressed proteins were associated with particle size [110]. In a recently published study [111], the same research group used these SNPs to study the global DNA methylation profiles in HaCaT cells; the authors reported that nanosilica treatment can induce epigenetic changes.

Cousins et al. [112] exposed murine fibroblasts to small **amorphous (colloidal) silica **particles (7, 14 and 21 nm) over a long incubation period (1, 3 and 7 days and up to 7 weeks) and observed a distinctive cellular response affecting the morphologic features, adhesion and proliferation of the fibroblasts but not cell viability. Chang et al. [113] exposed selected human fibroblast and cancer cell lines for 48 h to **amorphous **SNPs and assessed cellular viability by MTT and LDH assays. Cytotoxicity was seen at concentrations > 138 μg/ml and depended on the metabolic activity of the cell line. However, the average primary size of tested silica particles was 21 and 80 nm, but their average hydrodynamic particle size was 188 and 236 nm, respectively, so in media, aggregates/agglomerates were formed.

In the study of Yang et al. [114], cell membrane injury induced by 20-nm **amorphous silica **nanoparticles in mouse macrophages was closely associated with increased intracellular oxidative stress, decreased membrane fluidity, and perturbation of intracellular calcium homeostasis.

Besides inhalation, ingestion is considered a major uptake route of nanoparticles into the human body [3]; however, the possible harmful effects of engineered nanoparticles in the gastrointestinal tract are still largely unknown. Recently, Gerloff et al. [115] investigated the cytotoxic and DNA damaging properties of **amorphous **fumed SiO_2 _nanoparticles (14 nm) in the human colon epithelial cell-line Caco-2. Exposure to SNPs for up to 24 h caused cell mortality, significant DNA damage and total glutathione depletion. The results of an *in vivo *study of mice fed nanosized silica are discussed in section 3.2.2.

Ye et al. [116] reported on induced apoptosis in a human hepatic cell line after exposure to **amorphous (colloidal) **SNPs (21, 48 and 86 nm). The viability of cells was assessed by LDH and MTT assay; oxidative stress was studied by measurement of ROS, lipid peroxidation and GSH concentration; and apoptosis was quantified by annexin V/propidium iodide staining and DNA ladder assays. Nano-SiO_2 _caused cytotoxicity in a size-, dose- and time-dependent manner.

Because nanoparticles are probably distributed by the blood stream (e.g., with medical applications), endothelial cells would also come in direct contact with these particles, for pathogenic particle-endothelial interactions. Peters et al. [117] evaluated the effects of 4- to 40-nm **amorphous SiO_2 _**particles *in vitro *on human dermal microvascular endothelial cell function and viability. The particles were internalized but did not exert cytotoxic effects (MTS assay). However, cells showed impaired proliferative activity and pro-inflammatory stimulation. Napierska et al. [118] reported a dose-dependent cytotoxicity (by MTT and LDH assay) of **monodisperse amorphous **SNPs (16-335 nm) in a human endothelial cell line. The toxicity of the particles was strongly related to particle size; smaller particles showed significantly higher toxicity and also affected the exposed cells faster. Ye et al. [119] evaluated the toxicity of **amorphous **SNPs (21 and 48 nm) towards rat myocardial cells. Exposure to the SNPs for up to 48 h resulted in size-, dose- and time-dependent cytotoxicity, smaller particles again showing higher toxicity.

Barnes et al. [120] reported no detectable genotoxic activity (by Comet assay) of **amorphous **SNPs (20 nm to < 400 nm) in 3T3-L1 fibroblasts at 4 or 40 μg/ml silica for 24 h. The particle dispersions were carefully characterized and the results were independently validated in 2 separate laboratories. In a recent review, Gonzalez et al. [121], in a literature review, compared 2 genotoxicity tests -- the alkaline Comet assay and the micronucleus test - in terms of chemical composition and size of engineered SNPs: engineered SNPs did not seem to induce DNA strand breakage. However, when **monodisperse amorphous **SNPs of 3 different sizes (16, 60 and 104 nm) were selected to assess the genotoxic potential of these particles in A549 lung carcinoma cells with a well-validated assay (the *in vitro *cytochalasin-B micronucleus assay), at non-cytotoxic doses, the smallest particles showed an apparently higher-fold induction of micronucleated binucleated (MNBN) cells [122]. When considering the 3 SNPs together, particle number and total surface area accounted for MNBN induction because they were significantly associated with the amplitude of the effect.

#### Crystalline nanosilica

Wang et al. [99] investigated cytotoxicity (by MTT assay) and genotoxicity of ultrafine **crystalline SiO_2 _particulates **(UF-SiO_2_) in cultured human lymphoblastoid cells. A 24-h treatment with 120 μg/ml UF-SiO_2 _produced a fourfold increase in MNBN cells, with no significant difference as measured by the Comet assay. However, the ultrafine crystalline silica used was extracted from commercially available crystalline silica and the particle sizes were not uniform [99].

#### Mesoporous silica

The cytoxicity of **amorphous mesoporous SNPs **(**MSNs**) was recently studied intensively because they are promising materials for drug delivery systems and cell markers [8,123,124]. Several studies have demonstrated that efficient cellular uptake of MSNs could be achieved at concentrations < 50 μg/ml, with no cytotoxic effects observed up to 100 μg/ml in different mammalian cells [125-130]. Lu et al. [128] reported on the optimal size of ~50 nm MSNs for cell uptake. Slowing et al. [131] reported that, contrary to the known cytotoxicity of amorphous SNPs toward red blood cells, **mesoporous **SNPs exhibit high biocompatibility at concentrations adequate for potential pharmacological applications.

However, studies have reported cytotoxicity of mesoporous silica nanomaterials. Tao et al. [132] investigated the effects of two types of **MSNs **(pore diameters of 31 and 55 Å) on cellular bioenergetics (cellular respiration and ATP content) in myeloid and lymphoid cells and isolated mitochondria. Only cells exposed to MSNs with larger size and larger pores showed concentration- and time-dependent inhibition of cellular respiration, and both nanoparticles were toxic to the isolated mitochondria. Di Pasqua et al. [133] reported that the toxicity of **MSNs **towards human neuroblastoma cells was related to the adsorptive surface area of the particle. However, the nature of the functional groups playing a role could not be excluded. Vallhov et al. [134] investigated the effects of **mesoporous **SNPs of different sizes (270 nm and 2.5 μm) on human dendritic cells and found viability, uptake and immune regulatory markers affected by increasing size and dose. He et al. [135] evaluated the influence of size and concentration of **mesoporous **SNPs (190, 420 and 1220 nm) on cytotoxicity in human breast cancer cells and monkey kidney cells. The cytotoxicity of the particles was associated with particle size: silica of 190 and 420 nm in diameter showed significant cytotoxicity at concentrations > 25 μg/ml; whereas particles of 1220 nm in diameter showed slight cytotoxicity at 480 μg/ml. The smaller particles were suggested to be more easily endocytosed and consequently located within lysosomes [135].

#### Surface-modified/functionalized silica

Brown et al. [136] attempted to evaluate the role of shape in particle toxicity in the lung; the authors compared the response of rod-shaped and spherical **amorphous silica particles **(Stöber), not coated or coated with fibronectin or polyethylene glycol (PEG), under stretched and static conditions. The dosimetric comparison of materials with different shapes (e.g., needle-shaped or acicular and isotropic) was not straightforward. Non-coated particles induced an increase in IL-8 and LDH release, whereas a surface modification with PEG mitigated this effect, which suggested the significance of adhesive interactions for membrane binding/signal transduction, for example [136].

Diaz et al. [137] described the interactions of two **amorphous **silica particles - a pristine particle, without any coating, and PEGylated silica particles (average size 130 and 155 nm), as well as an iron oxide particle with a silica shell (80 nm) -- with different human peripheral blood cells, several human tumor cell lines and mouse peritoneal macrophages. The effects depended on the cell analyzed: although all particles were phagocytosed and were able to induce ROS expression in mouse macrophages, they differentially affected the human cell lines and peripheral blood cells, both in terms of internalization and ROS induction. The availability of the particles to be internalized by the cells seemed to strongly depend on aggregation, especially on the size and morphology of the aggregates [137].

Almost all of the existing cytotoxicity studies of SNPs involved monocultures of cells that are organ specific. The exception is the study by Wottrich et al. [103], in which co-cultures of epithelial cells (A549) and macrophages (THP-1, Mono Mac 6) exposed to 60- and 100-nm amorphous SNPs showed an increased sensitivity to the cytokine release as compared with monocultures of each cell type. The enhanced responses to nanoparticles in different contact and non-contact co-cultures were reported in studies by Herseth et al. [138,139] with micron-sized crystalline silica, showing that more realistic models should be applied to study interactions between nanoparticles and cells or organs of interest.

Few recently published studies have systematically investigated nanomaterial properties in terms of the degree and pathways of cytotoxicity. Sohaebuddin et al. [140] selected nanomaterials of different composition, including silica, to analyze the effects of size and composition on 3 model cell lines: fibroblasts, macrophages and bronchiolar epithelial cells. The authors concluded that the physico-chemical properties of size and composition both determined the cellular responses and induced cell-specific responses. In another recent study, Rabolli et al. [141] studied the influence of size, surface area and microporosity on the *in vitro *cytotoxic activity of a set of 17 stable suspensions of **monodisperse amorphous **SNPs of different sizes (2-335 nm) in 4 different cell types (macrophages, fibroblasts, endothelial cells and erythrocytes). The response to these nanoparticles was governed by different physico-chemical parameters that varied by cell type: in murine macrophages, the cytotoxic response increased with external surface area and decreased with micopore volume; in human endothelial cells and mouse embryo fibroblasts, the cytotoxicity increased with surface roughness and decrease in diameter; and in human erythrocytes, the hemolytic activity increased with the diameter of the SNP [141].

Overall, most of these *in vitro *studies involving different SNPs documented the cytotoxic effects of these nanomaterials. The determinants of the observed cytotoxicity seem to be complex and vary with the particles used and cell type tested. Unfortunately, for many published studies, adequate material characterization is still missing. The mere cytotoxicity reported with some particles does not strictly imply hazard. However, this observation indicates that a proactive development of nanomaterials should consider physical, chemical and catalytic properties of nanoparticles.

### *In vivo *studies of nanosilica toxicity

Along with particle size, surface area and particle number appear to be integral components contributing to the mechanisms of lung toxicity induced by nano-sized particles. The high deposition rate of ultrafine particulates is a result of a small aerodynamic diameter and is assumed to be important in the lung inflammatory process. Some evidence suggests that inhaled nanoparticles, after deposition in the lung, largely escape from alveolar macrophage clearance and gain greater access to the pulmonary interstitium via translocation from alveolar spaces through epithelium [3,142]. A summary of the *in vivo *responses to SNPs can be found in Table 3.

**Table 3:** In vivo studies on nanosilica particles (SNPs) toxicity

Silica form	Size (primary)	Material characterization	Exposure model	Test	Biological endpoints and findings	Ref
Quartz	10-20 nm (average size: 12), 30-65 (average size: 50), 300 nm - 2 μm	• Synthesis • Surface area • Crystallinity • Metal impurities	Rats instilled intratracheally with various particle types (1 or 5 mg/kg), sacrificed at 24 h, 1 week, 1 month, and 3 months post-exposure	• Bronchoalveolar lavage (BAL) fluid analysis: cell counts, differentials, and pulmonary biomarkers (Lactate dehydrogenase (LDH), alkaline phosphatase (ALP), and lavage fluid protein) • Cell proliferation • Morphological/Histopathology examination • Hemolytic Potential of particles	Exposures to the various quartz particles produced differential degrees of pulmonary inflammation and cytotoxicity, which were not consistent with particle size but correlated with surface activity, particularly hemolytic potential.	[148]

Silica dust	10 ± 5 nm; and 0.5-10 μm (80% of the particles 1-5 μm)	• Composition uknown • Surface area	Rats instilled intratracheally (20 mg), sacrificed 1 and 2 months after dosing	• The changes of lung/body coefficient and hydroxyproline content • Pathologic examination • Immunohistochemical staining for IL-4 and TGF-beta1	One month after instillation cellular nodules (Stage I silicosis) were found in the nanosized SiO_2 _group, while in microsized SiO_2 _group Stage II, II+ of silicotic nodules were observed. Two months after instillation, still only Stage I silicotic nodules in nanosilica group were found, while in the micro-silica group the disease progressed and Stage II+, and III silicotic nodules were found. The experiment revealed that in rats the effect of fibrogenesis of nano-SiO_2 _might be milder than that of micro-SiO_2_.	[147]

Ludox colloidal silica	-	• Mass median aerodynamic diameter (2.9, 3.3 and 3.7 μm) • Chamber Ludox concentration	Rats Inhalation (nose-only) for 2 or 4 weeks at concentrations 10, 50 and 150 mg/m^3^. Additional groups of rats exposed for 4 weeks were given a 3-month recovery period	• Lung silica analysis • BAL analysis: cell differential counts and biochemical assay (LDH, ALP, lavage fluid protein) • Pulmonary macrophage cell culture and phagocytosis assay • SEM ananlysis • Additional groups of animals were processed for cell labeling studies or lung deposition studies.	The inflammatory responses, mainly seen as increased numbers of neutrophils in BALF, following the 2 and/or 4 weeks of exposure was evident at 50 mg/m^3 ^(or higher) group. Three months after exposure most biochemical parameters returned to control values. Results showed that exposures to 150 mg/m3 Ludox for 2 or 4 weeks produced pulmonary inflammation along with increases in BAL protein, LDH, and alkaline phosphatase values (p less than 0.05) and reduced macrophage phagocytosis. Autoradiographic studies demonstrated that the labeling indices of terminal bronchiolar and lung parenchymal cells were generally increased in the 50 and 150 mg/m3 groups after 2 and 4 weeks of exposure but, with one exception, returned to normal levels following a 3-month postexposure period.	[143]

Aerosol containing colloidal silica	Average size: 22 nm	• Mass median aerodynamic diameter (2.9, 3.3 and 3.7 μm) • Chamber Ludox concentration	Rats inhalation (from 10 to 150 mg/m^3^), 6 h/day, 5 days/week for 4 weeks; 3 months postexposure	• Lung silica determination • Body weights and clinical observations • Clinical pathology (urine and blood samples) • Histopathology	No effects after exposure to the lowest concentration Lung weights were increased significantly after 4 exposure to 50 and 150 mg/m^3^. A dose dependent alveolar macrophage response, polymorphonuclear leukocytic infiltration, and Type II pneumocyte hyperplasia in alveolar duct regions was reported. Lung-deposited nanosilica cleared rapidly from the lungs with half-times of approximately 40 and 50 days for the 50 and 150 mg/m^3 ^groups, respectively. The lungs did not show fibrotic scar tissue formation or alveolar bronchiolarization.	[52]

Colloidal silica	(UFCSs, average size of 14 nm) fine colloidal silica particles (FCSs; average size of 213 nm)	• Size distribution • Surface area • Metal composition	Mice instilled intratracheally (3 mg) and sacrificed 0.5, 2, 6,12 and 24 h after dosing	• Histopathology • Immunohistochemistry • Electron microscopy	Histopathological examination revealed for both sizes bronchiolar degeneration, necrosis, neutrophilic inflammation, alveolar type II cell swelling and alveolar macrophage accumulation. UFCs induced extensive alveolar hemorrhage, a more severe bronchiolar epithelial cell necrosis and neutrophil influx in alveoli compared to FCSs. Electron microscopy demonstrated UFCSs and FCSs on bronchiolar and alveolar wall surface as well as in the cytoplasm of alveolar epithelial cells, alveolar macrophages and neutrophils. The findings suggest that UFCSs (possibly linked to larger surface area) have greater ability to induce lung inflammation and tissue damages than FCSs.	[98]

Colloidal silica	average size: 14 nm	• Size distribution • Surface area • Metal composition	Mice instilled intratracheally (0.3,3,10,30 or 100 μg) and sacrificed 3 days after dosing; 1 to 30 days postexposure	• BAL analysis: cells quantification, viability and differentiation, total protein concentration • Histopathology • Immunohistochemistry • Apoptosis (TUNEL assay)	Exposure up to 100 μg of UFCSs produced moderate to severe pulmonary inflammation and tissue injury 3 days post exposure. Mice instilled with 30 μg of UFCSs and sacrificed at intervals from 1 to 30 days post-exposure showed moderate pulmonary inflammation and injury on BALF indices at acute period; however, these changes gradually regressed with time. Histopathological and immunohistochemical examination correlated to BALF data. A significant increase of the apoptotic index (TUNEL) in lung parenchyma at all observation times was reported. The findings suggest that instillation of a small dose of UFCSs caused an acute, but transient, lung inflammation and tissue damage in which oxidative stress and apoptosis may be involved.	[146]

Amorphous silica	14 nm	• Endotoxins content	Mice instilled intratracheally (2,10 and 50 mg/kg) and sacrificed 24 h, 1,4 and 14 weeks after dosing	• BAL analysis: total protein and endotoxin concentration, cell differential counts • Histopathology • Real-time PCR • Immunohistochemistry	Significantly increased lung weights, total BAL cells and proteins were observed until 1 week after treatment. Particles induced acute inflammation (with neutrophils) at an early stage and chronic granulomatous inflammation at the later stage. The significant up-regulation of cytokines (IL-1β, IL-6, IL-8, and TNF-α) and chemokines (MCP-1 and MIP-2) was observed during the early stages, but there were no changes after week 1. In conclusion, Instillation of nanoparticles induced transient but very severe lung inflammation.	[144]

Amorphous silica	37.9 ± 3.3 nm	• Size distribution • Surface area • Particle number	Rats inhalation (24.1 mg/m^3^, 40 min/day, 4 weeks The age factor involved 3 levels (young/ adult/old)	• Electrocardiography • BAL analysis • Hemorheological analysis • Serum biomarker assay • Pathology	Inhalation of SNP under identical conditions caused the strongest pulmonary and cardi ovascular alterations in old rats, yet less change in young and adult rats. Observed changes included pulmonary inflammation, myocardial ischemic damage, atrio-ventricular blockage, and increase in fibrinogen concentration and blood viscosity. Old individuals were more sensitive to nanoparticle exposure than the young and adult rats. The risk of causing pulmonary damages was: old > young > adult. The risk of cardiovascular disorder was observed only in old age.	[145]

Amorphous silica	37 nm and 83 nm	• The generation of nanosilica aerosol • Size distribution	Rats inhalation (3.7 × 10^7 ^or 1.8 × 10^8 ^particles/cm^3^), 6h/day, for 1- or 3-days several post-exposure time points (up to 2 months)	• Bal analysis: cell counts, differentials, enzymatic activity of LDH,and ALP • Genotoxicity endpoints (micronuclei induction)	One- or three-day aerosol exposure produced no significant pulmonary inflammatory, genotoxic, or adverse lung histopathological effects in rats exposed to very high particle numbers corresponding to a range of mass concentrations (1.8 or 86 mg/m^3^).	[149]

Amorphous silica	14 nm	o Daily mean mass median aerodynamic diameter (2.1 ± 0.1 μm)	Rats inhalation (head/nose only; 26.9 ± 3 mg/m^3^), 6h/day during 6 days); Challenging the animals by inhalation to a minimally irritating concentration of allergen trimellitic anhydride (TMA)	• Breathing parameters • Cellular and biochemical changes in BAL • Histopathological airway changes	Exposure to SNPs alone resulted in transient changes in breathing parameters during exposure, and in nasal and alveolar inflammation with neutrophils and macrophages. Exposure to particles before a single TMA challenge resulted in only a slightly irregular breathing pattern during TMA challenge. Pre-exposure to particles also diminished the effect of TMA on tidal volume, laryngeal ulceration, laryngeal inflammation, and the number of BAL eosinophils in most animals. When the additional group of animals was exposed to nanosilica before a second challenge to TMA, the pulmonary eosinophilic infiltrate and edema induced by a second TMA challenge in control animals was diminished by the preceding silica exposure, but the number of lymphocytes in BAL was increased.	[153]

Amorphous silica	~30 nm and ~30 μm	• Size distribution	Feeding of mice for 10 weeks (total fed amount of 140 g/kg mice)	• Blood analysis • Cytological analysis of lungs and liver tissue sections • Analysis of silicon in organs	The nano-sized silica particle dieted group showed higher value of ALT (alanine aminotransferase) than normal and micron-sized silica dieted groups. H&E staining of the liver of the nano-sized particle dieted group indicated some fatty liver pattern. The contents of Si in the livers of the groups were almost the same.	[154]

Amorphous silica (organically modified)	20-25 nm	• Synthesis • Conjugation with fluorophore • Radiolabelling	Mice injected intravenously with SPN (2.0 mg/kg body weight)	• Fluorescence imaging (CRi) • MicroPET imaging • Histological Analysis	Greater acummulation of nanoparticles in liver, spleen and stomach that in kidney, heart and lungs. Almost 100% of the injected nanoparticles were effectively cleared out of the animals over a period of 15 days via the hepatobiliary excretion. No signs of organs toxicity were observed.	[155]

Amorphous (mesoporous) silica	150 nm, 800 nm and 4 μm (pore sizes of 3 nm, 7 nm and 16 nm)	• Synthesis • Size • Endotoxins content	Rats injected subcutaneously (30 mg per rat), Mice injected intraperitoneally and intravenously	• Hematoxylin and eosin staining and histological examination	When the particles were injected subcutaneously, the amount of residual material decreased progressively over 3 months, with good biocompatibility on histology at all time points. Intra-peritoneal and intra-venous injections in mice resulted in death or euthanasia. No toxicity was seen with subcutaneous injection of the same particles in mice. Microscopic analysis of the lung tissue of the mice indicated that death may be due to thrombosis.	[156]

Amorphous silica	75, 311 and 830 nm	Not specified	Mice injected intravenously (10-100 mg/kg)	• H&E staining; histological analysis of the liver, kidney, spleen and lung • Biochemical assays • Gadolinium chloride, cyclophosphamide and hepatic hydroxyproline assay	70 nm SNP induced liver injury at 30 mg/kg body weight, while SP300 or 1000 had no effect even at 100 mg/kg. Administration of 70 nm SNP dose-dependently increased serum markers of liver injury, serum aminotransferase and inflammatory cytokines. Repeated administration of 70 nm SNP twice a week for 4 weeks, even at 10 mg/kg, caused hepatic fibrosis.	[157]

Amorphous silica	50, 100 and 200 nm	• Synthesis • Fluorescence labeling	Mice injected intravenously (50 mg/kg)	• Confocal laser scanning microscopy • Immunofluorescence staining • Fluorescence microplate readings	Significant increase of inflammation in the liver at 12 h for the 100 and 200 nm silica nanoparticles treatment groups. The tissue distribution and excretion of the injected particles were different depending on particle size. As particle sizes increased, more particles were trapped by macrophages in the liver and spleen. All particles were cleared via urine and bile; however, the 50 nm silica nanoparticles excreted faster than the other two particles.	[158]

In 1991, Warheit et al. [143] performed a rat inhalation study (nose-only) with an **aerosol of colloidal silica **(mass median aerodynamic diameter 2.9, 3.3 and 3.7 μm) for 2 or 4 weeks at concentrations up to 150 mg/m^3^, and some groups of rats were allowed to recover for 3 months. The inflammatory responses, mainly seen as increased numbers of neutrophils in bronchoalveolar lavage fluid (BALF), with the 2 and/or 4 weeks of exposure were evident at ≥ 50 mg/m^3 ^concentration. Three months after exposure, most biochemical parameters returned to control values [143].

Lee and Kelly [52] studied the effects of repeated inhalation (6 h/day, 5 days/week for 4 weeks) of an **aerosol of colloidal silica **(mass median aerodynamic diameter 2.9, 3.3 and 3.7 μm; concentration up to 150 mg/m^3^) in rats. The authors reported a dose-dependent alveolar macrophage response, polymorphonuclear leukocytic infiltration, and type II pneumocyte hyperplasia in alveolar duct regions. Lung-deposited nanosilica were cleared rapidly from the lungs, with half-times of approximately 40 and 50 days for the 50 and 150 mg/m^3 ^treatment groups, respectively. The lungs did not show formation of fibrotic scar tissue or alveolar bronchiolarization [52].

Cho et al. [144] investigated inflammatory mediators (24 h, and 1, 4 or 14 weeks after exposure) induced by intratracheal instillation in mice of up to 50 mg/kg of ultrafine **amorphous silica **with a primary particle diameter of 14 nm. The authors observed significantly increased lung weights, total cell numbers and levels of total protein in BALF up to 1 week after treatment. The histopathological examination revealed acute inflammation, with neutrophils and chronic granulomatous inflammation. The expression of cytokines (IL-1β, IL-6, IL-8, and TNF-α) and chemokines (monocyte chemoattractant protein 1 and macrophage inflammatory protein 2) was significantly increased during the early stages, with no changes after week 1 [144].

Chen et al. [145] studied age-related differences in response to **amorphous **SNPs (average size 38 nm). Changes in serum biomarkers, pulmonary inflammation, heart injury and pathology were compared in young (3 weeks), adult (8 weeks) and old (20 months) rats that inhaled tested nanoparticles for 4 weeks (40 min/day). Old animals appeared to be more sensitive to nanoparticle exposure than were young and adult rats. The risk of pulmonary damage was old > young > adult, but the risk of cardiovascular disorder was observed only in old animals [145].

Kaewamatawong et al. [98] compared acute pulmonary toxicity induced in mice by ultrafine **colloidal silica **particles (UFCSs; average size 14 nm) or fine colloidal silica particles (FCSs; average size 213 nm) after intratracheal instillation of 3-mg particles. Histopathological examination with both sizes revealed bronchiolar degeneration, necrosis, neutrophilic inflammation, alveolar type II cell swelling and alveolar macrophage accumulation. However, UFCSs induced extensive alveolar hemorrhage, more severe bronchiolar epithelial cell necrosis and neutrophil influx in alveoli as compared with FCSs. Electron microscopy showed UFCSs and FCSs on the bronchiolar and alveolar wall surface and in the cytoplasm of alveolar epithelial cells, alveolar macrophages and neutrophils. The findings suggest that UFCSs (possibly linked to size and/or larger surface area) have a greater ability to induce lung inflammation and tissue damage than do FCSs [98]. The same research group reported acute and subacute pulmonary toxicity of low-dose UFCS particles in mice after intratracheal instillation [146]. Exposure of up to 100 μg UFCSs produced moderate to severe pulmonary inflammation and tissue injury 3 days after exposure. Mice instilled with 30 μg UFCSs and sacrificed at intervals from 1 to 30 days after exposure showed moderate pulmonary inflammation and injury on BALF indices at the acute period; however, these changes gradually regressed with time. Concomitant histopathological and laminin immunohistochemical results were similar to BALF data. The authors reported a significant increase in the apoptotic index (TUNEL) in lung parenchyma at all observation times. The findings suggest that instillation of a small dose of UFCSs causes acute but transient lung inflammation and tissue damage in which oxidative stress and apoptosis may be involved [146].

In a study of fibrogenesis, Wistar rats were intratracheally instilled with silica (of unknown composition) nano- (10 ± 5 nm) and microparticles (0.5-10 μm), and were sacrificed 1 and 2 months after dosing [147]. One month after instillation, cellular nodules (Stage I silicosis) were found in the nano-sized SiO_2 _group, whereas more severe lesions were found in the micron-sized SiO_2 _treatment group (Stage II and Stage II+ of silicotic nodules). One month later, the nano-sized SiO_2 _group still showed only Stage I silicotic nodules, whereas the micron-silica group showed disease progression and Stage II+ and III silicotic nodules. Therefore, in rats, the effect of nano-SiO_2 _on fibrogenesis might be milder than that of micron-SiO_2_. Nanoparticles, because of their size, probably diffuse more easily to other pulmonary compartments than do microparticles [147].

Warheit et al. [148] compared the toxicity of synthetic **nanoquartz **particles (12 and 50 nm) to mined Min-U-Sil quartz (500 nm) and synthetic fine-quartz particles (300 nm) and (2) evaluated the surface activity (hemolytic potential) of the different samples in terms of toxicity. Rats were instilled with the different particle types (1 or 5 mg/kg), and pulmonary toxicity was assessed with BALF biomarkers, cell proliferation, and histopathological evaluation of lung tissue at 24 h, 1 week, 1 month, and 3 months after exposure. Exposure to the quartz particles of different sizes produced pulmonary inflammation and cytotoxicity, with nanoscale quartz of 12 nm and Min-U-Sil quartz being more toxic than fine quartz and nanoscale quartz of 50 nm. The pulmonary effects were not consistent with particle size but were associated with surface activity, particularly hemolytic potential [148].

In a recent work by Sayes et al. [149], rats inhaled freshly generated aerosolized **amorphous **SNPs of 37 and 83 nm for a short-term period. In contrast to previous studies' measurements, particle number rather than particle mass was chosen as dose metrics (3.7 × 10^7 ^or 1.8 × 10^8 ^particles/cm^3^) for 1- or 3-day exposure. Pulmonary toxicity (cell counts, differentials, enzymatic activity of LDH and alkaline phosphatase (ALP) in BALF) and genotoxicity endpoints (micronuclei induction) were assessed from 24 h up to 2 months after exposure. One- or 3-day aerosol exposure produced no significant pulmonary inflammatory, genotoxic or adverse lung histopathological effects in rats exposed to very high particle numbers in a range of mass concentrations (1.8 or 86 mg/m^3^).

Recently, airway irritants were suggested to facilitate allergic sensitization [150-152]. Arts et al. [153] examined the effect of pre-exposure to synthetic (fumed) **amorphous **SNPs (14 nm) on elicitation of airway hypersensitivity reactions by the low-molecular-weight allergen trimellitic anhydride (TMA). Brown Norway rats were topically sensitized with TMA, exposed (head or nose only) to SNPs for 6 h/day for 6 days and then challenged by inhalation with a minimally irritating concentration of TMA. One day later, breathing parameters, cellular and biochemical changes in BALF, and histopathological airway changes were studied. Exposure to SNPs alone resulted in transient changes in breathing parameters during exposure and in nasal and alveolar inflammation with neutrophils and macrophages. Exposure to particles before a single TMA challenge resulted in only a slightly irregular breathing pattern during TMA challenge. Interestingly, pre-exposure to particles diminished the effect of TMA on tidal volume, laryngeal ulceration, laryngeal inflammation, and the number of BALF eosinophils in most animals. When an additional group of animals was exposed to nanosilica before a second challenge to TMA, the pulmonary eosinophilic infiltrate and edema induced by a second TMA challenge in control animals was diminished by the preceding silica exposure, but the number of lymphocytes in the BALF was increased. The authors concluded that SNPs could reduce as well as aggravate certain aspects of TMA-induced respiratory allergy [153].

As mentioned, next to inhalation, ingestion is considered a major route for the uptake of nanoparticles in the human body. So et al. [154] studied the effects on mice fed nano- and micron-sized **amorphous **silica particles (30 nm and approximately 30 μm, respectively). After feeding the animals for 10 weeks (total amount of 140 g silica/kg mouse), blood was tested biochemically and hematologically. The group fed SNPs showed higher serum values of alanine aminotransferase as compared with the other groups (both control and micron-silica treated). Although the contents of Si in the livers of the groups were almost the same, hematoxylin and eosin staining revealed a fatty liver pattern in the group treated with SNPs [154].

The successful use of nanoparticles in the clinic requires exhaustive studies on the behavior of these particles *in vivo*. Unfortunately, biocompatibility, biodistribution and clearance studies of silica-based nanoparticles are sparse. Kumar et al. [155] used nanoparticles of organically modified **amorphous **silica (ORMOSIL; amino-terminated; 20-25 nm) to study biodistribution, clearance and toxicity in a mouse model. Particles conjugated with fluorophore and radiolabeled were injected systemically in mice. Biodistribution studies showed a greater accumulation of nanoparticles in liver, spleen and stomach than in kidney, heart and lungs. Over 15 days, almost 100% of the injected nanoparticles were effectively cleared out of the animals via hepatobiliary excretion, without any sign of organ toxicity. Hudson et al. [156] examined the biocompatibility of **mesoporous **silica particles (150 nm, 800 nm and 4 μm) after injection in rats and mice. When the particles were injected subcutaneously in rats, the amount of residual material decreased progressively over 3 months, with no significant injury to surrounding tissues. Subcutaneous injection of the same particles in mice produced no toxic effects. In contrast, intra-peritoneal and intra-venous injection in mice resulted in death; microscopic analysis of the lung tissue of the mice indicated that death might have been due to pulmonary thrombosis. Nishimori et al. [157] evaluated the acute toxicity of **amorphous **silica particles (70, 300 and 1000 nm) after a single intravenous injection in mice and reported that 70-nm silica injured the liver but not the spleen, lung or kidney. Moreover, chronic administration of 70-nm nanoparticles (injections every 3 days for 4 weeks) caused liver fibrosis. Cho et al. [158] examined the impact of the size of **amorphous **SNPs on toxicity, tissue distribution and excretion. Fluorescence dye-labeled 50-, 100- and 200-nm silica particles were intravenously injected in mice. The incidence and severity of inflammation with the 100- and 200-nm SNPs was significantly increased in the liver at 12 h; the 50-nm particles induced a slight but nonsignificant inflammatory response. The tissue distribution and excretion of the injected particles differed depending on particle size. With increasing particle size, more particles were trapped by macrophages in the liver and spleen. All particles were cleared via urine and bile; however, the 50-nm SNPs were excreted faster than were the other 2 particle sizes [158].

### In *vivo *versus *in vitro*; amorphous versus crystalline

Park and Park [159] performed *in vitro *and *in vivo *studies to investigate oxidative stress and pro-inflammatory responses induced by **amorphous **SNPs (average primary size 12 nm). RAW 264.7 cells derived from mouse peritoneal macrophages were exposed to SNPs (5-40 ppm) *in vitro *and showed ROS generation and decreased intracellular GSH levels, as well as increased levels of nitric oxide released from the cultured macrophage cell line. *In vivo*, mice were treated with a single intraperitoneal dose of 50 mg/kg of nanosilica. The treatment produced activated peritoneal macrophages, increased blood level of IL-1β and TNF-α, and increased level of nitric oxide released from peritoneal macrophages. *Ex vivo*, cultured peritoneal macrophages harvested from the treated mice showed the expression of inflammation-related genes (IL-1, IL-6, TNF- α, inducible nitric oxide synthase, cyclooxygenase 2). In the spleen, the relative distribution of natural killer cells and T cells was increased 184.8% and 115.1%, respectively, as compared with control animals, and that of B cells was decreased to 87.7% [159].

Kim et al. [160] addressed the toxicity of nano- and micron-sized silica particles (14 nm and 1-5 μm, respectively) *in vitro *and *in vivo. In vitro*, RAW 264.7 cells were exposed to both particle sizes for 24 h, and the cell viability was decreased in dose-dependent manner; however, apoptosis was observed only after treatment with nanoparticles. *In vivo*, mice received up to 5 mg/kg silica particles via oropharyngeal aspiration. Again, size-dependent toxicity of silica was observed; pulmonary injury and neutrophilic infiltration were greater after treatment with nano-sized SiO_2 _particles than with micron-sized silica [160].

Sayes et al. [161] assessed the capacity of *in vitro *screening studies to predict *in vivo *pulmonary toxicity of several fine or nanoscale particle types in rats. For the *in vitro *component of the study, rat lung epithelial cells, primary alveolar macrophages and alveolar macrophages-lung epithelial cell co-cultures were incubated with **quartz **particles and precipitated **amorphous **silica. In the *in vivo *component of the study, rats were exposed by intratracheal instillation to the same particles. *In vivo*, pulmonary toxicity studies demonstrated that crystalline silica particles produced sustained inflammation and cytotoxicity, whereas amorphous silica particles produced reversible and transient inflammatory responses. *Ex vivo*, pulmonary inflammation studies showed that crystalline and amorphous silica-exposed rat lung epithelial cells did not produce MIP-2 cytokines, but alveolar macrophages and, to a lesser degree, co-cultures secreted this chemotactic factor into the culture media. *In vitro *cytotoxicity studies demonstrated a variety of responses to the different particle types, primarily at high doses. When considering the range of toxicological endpoints, comparisons of *in vivo *and *in vitro *measurements revealed little correlation, particularly when considering the many variables assessed in this study such as cell types used, culture conditions and time course of exposure, as well as measured endpoints.

To summarize, extrapolating (or comparing) the results obtained *in vitro *to the *in vivo *situation is difficult and applies not only to toxicity studies with nanoparticles -- any existing *in vitro *test system lacks the complexity of animal models or the human body. However, considering the number of particles and the number of possible properties of these particles that may vary (size, shape, coating, etc.), clearly, not all can be evaluated in *in vivo *studies, and scientists have been striving to determine the correlation between the results obtained from *in vitro *and *in vivo *toxicity assessments. Although little correlation has been found in these studies with nanosilica [159-161], Lu et al. [162] tested a panel of metal oxide nanoparticles and could predict the inflammogenicity of tested nanomaterials with a battery of simple *in vitro *tests. Similar conclusions were drawn in a recent study by Rushton et al. [163]; the authors could predict the acute *in vivo *inflammatory potential of nanoparticles with cell-free and cellular assays by using NP surface area-based dose and response metrics. The authors also found that a cellular component was required to achieve a higher degree of predictive power.

Established and validated co-culture systems may provide a tool to better mimic the *in vivo *system. Using recently developed 3-D cell cultures and improving the exposure system (likewise exposure at the air-liquid interface of a human epithelial airway model reported by Brandenberger et al. [164]), could substantially improve the outcome from *in vitro *studies with nanomaterials.

## Conclusions

Silica or silicon dioxide (SiO_2_) is, in many forms, abundantly present in our natural environment. The adverse health effects, including lung cancer, of naturally occurring crystalline silica such as quartz and cristobalite have been thoroughly documented in occupational settings. Naturally occurring amorphous silica such as diatomaceous earth is considered less harmful. Most of the synthetic (manufactured) silicas used in a large variety of applications are amorphous. For silica in general, the property most significantly linked to the toxicological potential is the crystallinity. For micron-sized crystalline silica, oxidative stress and, linked to it, oxidative DNA and membrane damage, are probably the most important mechanisms involved in the inflammogenic and fibrogenic activities (reviewed by [60]) and/or carcinogenic activity [39,165], for example. These mechanisms do not apply to amorphous silica, which has therefore been far less studied. Moreover, the adverse health effects of biogenic (natural) amorphous silica is often attributed to a certain degree of contamination with crystalline silica [49]. Synthetic amorphous silica (colloidal silica, fumed silica and precipitated silica) is not involved in progressive fibrosis of the lung [52,53]; however, high doses of amorphous silica may result in acute pulmonary inflammatory responses [54].

Interest in using SNPs is growing worldwide, especially for biomedical and biotechnological applications such as cancer therapy, DNA transfection, drug delivery, and enzyme immobilization [5-9]. In general, SNPs are synthetic, which has an advantage over natural silica in that they contain fewer or no impurities than do natural silica, and the physico-chemical properties are known and well controlled during production. Exposure to SNPs during the production process and their downstream use is probably minimal for sols and gels because the nanoparticles are trapped/immobilized within their matrix. However, the inhalation potential of low-density fumed silica powders or freeze-dried nanoparticles may be high without adequate precautions.

Results of a growing number of *in vitro *studies indicate that the particle surface area may play a crucial role in the toxicity of silica [75,166]. The cytotoxic activity of silica particles can be related to their surface interfacing with the biological milieu rather than to particle size or shape [75]. Surface silanol groups are directly involved (as shown *in vitro*) in hemolysis [76-78] and in alveolar epithelial cell toxicity [79,80]. This observation indirectly links the hydrophilicity to cellular toxicity [80,81]. The size and surface physico-chemical features of SNPs contribute decisively to the biological effects of SiO_2 _nanoparticles. The complexity of protein-SNP interactions should not be underestimated; these interactions appear to be affected by the size of SNPs as well [167-171]. The effect of other physico-chemical properties of SNPs on health, such as porosity, chemical purity, surface chemistry and solubility, are less well studied, and therefore no definite conclusions can be formulated (summary of the data can be found in Table 2). Comparison of published studies leads to the conclusion that even a small modification of the surface can result in a more or less marked change of a biological effect [2,3,172]. Few *in vitro *studies have emphasized that the response to SNPs varies by cell type [137,140,141].

Considering the use of SNPs for medical applications, biocompatibility and toxicokinetics need to be documented in great detail because, despite no observation of acute (cyto)toxicity, the uptake of the particles by cells may eventually lead to perturbation of intracellular mechanisms. For instance, the ability of silica-coated nanomaterials to penetrate the blood-brain barrier supports the urgent need for extensive studies to clarify the potential chronic toxicity of these materials [14]. The successful use of nanoparticles in the clinic requires exhaustive and elaborate *in vivo *studies [155]. Of note, the toxicity of SNPs can depend on not only the material itself but also the administration route to the living body, as was shown by Hudson et al. [156]: subcutaneous injection presented good biocompatibility, whereas intraperitoneal and intravenous injection led to fatal outcomes.

Unfortunately, only limited short-term and no chronic *in vivo *studies of SNPs are available (summary of the data is found in Table 3), and the current data do not clarify whether amorphous SNPs - showing augmented cytotoxicity and presumably processing oxidative DNA damaging potential -- are less or more harmful as compared with micron-sized silica.

Determining the association of results from *in vitro *and *in vivo *toxicity assessments is difficult; however, the common feature seems to be cytotoxicity and inflammatory response after exposure to SNPs.

To conclude, the available studies of the toxicity of SNPs are relatively few, especially as compared to the vast number of studies of titanium dioxide or carbon nanotubes. Besides the relative lack of information on the safety or hazards of SNPs, often conflicting evidence is emerging in the literature as a result of a general lack of standard procedures, as well as insufficient characterization of nanomaterials in biological systems. For all studies, a crucial issue remains the careful, accurate characterization of particle size and morphologic features (especially in the biological media used for experimental set-up), composition, particle surface area and surface chemistry [173]. Moreover, equally important to the physico-chemical characterization of the material is the control of assays and assay conditions [174,175]. Only with the complete description of the NP and assay can the results of reported studies be comparable with those of other studies conducted with similar nanomaterials [159,176].

Until now, the health effects of SNPs have mainly been studied in terms of exposure via the respiratory tract, after acute or sub-acute exposure; other exposure routes should also be checked (e.g. blood, skin, gastrointestinal tract). Studies of chronicity are needed to supplement and verify the existing data. Information is insufficient to clearly identify and characterize the health hazards SNPs pose, and defining the appropriate conditions for safe use of these materials is currently not possible.

## Competing interests

The authors declare that they have no competing interests.

## Authors' contributions

DN, LCJT and PHH drafted the manuscript. DN provided key input in the literature search. LCJT and JAM wrote the section on synthesis and characterization of silica materials. LCJT prepared all figures. DL contributed to drafting the paper. All authors read and approved the final manuscript.
